# Explainable Classification Models for Identifying Elevated Gastrointestinal Symptom Burden in Honduran Communities: A Cross-Sectional Study

**DOI:** 10.3390/diagnostics16213440

**Published:** 2026-10-22

**Authors:** Ethel Flores, Diana Meza, Rene Gonzales, Carlos Agudelo-Santos, Lilian Sosa, Lupe Carolina Espinoza, Isaac Zablah

**Affiliations:** 1 Faculty of Medical Sciences, National Autonomous University of Honduras, Calle la Salud, Tegucigalpa 11101, Honduras; ethel.flores@unah.edu.hn (E.F.); rene.gonzales@unah.edu.hn (R.G.); 2 Center for Biomedical Imaging Diagnostics Research and Rehabilitation, National Autonomous University of Honduras, Boulevard Suyapa, Tegucigalpa 11101, Honduras; carlos.agudelo@unah.edu.hn; 3 Universidad Pedagógica Nacional Francisco Morazán, Colonia Miraflores, Tegucigalpa 11101, Honduras; liliansosa2012@gmail.com; 4 Instituto de Investigaciones en Microbiología (IIM), Facultad de Ciencias, Universidad Nacional Autónoma de Honduras, Tegucigalpa 11101, Honduras; 5 Grupo de Investigación en Tecnología Farmacéutica, Facultad de Ciencias Químicas y Farmacia, Universidad Nacional Autónoma de Honduras, Tegucigalpa 11101, Honduras; 6 Departamento de Química, Facultad de Ciencias Exactas y Naturales, Universidad Técnica Particular de Loja, San Cayetano Alto, Loja 1101608, Ecuador; lcespinoza@utpl.edu.ec

**Keywords:** artificial intelligence, machine learning, explainable artificial intelligence, gastrointestinal symptoms, clinical decision support, primary health care, health equity, Honduras

## Abstract

**Background/Objectives:** Gastrointestinal symptoms are a common reason for primary-care consultation, yet specialist assessment is often inaccessible in rural Central America. We evaluated whether explainable models using inexpensive demographic and lifestyle data can identify community residents aged 12 to 49 years with concurrently elevated gastrointestinal symptom burden. **Methods:** This was a cross-sectional survey of 1838 residents from 85 communities in five Honduran departments. Elevated symptom burden was defined as at least two of four indicators: recent abdominal pain, heartburn, gastroesophageal reflux, and post-prandial bloating. Model A used eight demographic and lifestyle predictors; Model B added self-reported previous digestive diagnosis. Logistic regression was compared with six machine-learning classifiers under repeated nested five-fold and ten-fold cross-validation and community-grouped cross-validation. Discrimination, calibration, paired differences, community-level uncertainty, decision curves, SHAP explainability and subgroup performance were assessed. **Results:** Elevated symptom burden affected 840/1838 participants (45.7%, 95% CI 43.4 to 48.0). Model A showed modest discrimination (AUROC 0.663, 95% CI 0.639 to 0.688), while Model B improved performance (AUROC 0.726, 95% CI 0.702 to 0.748) with good calibration (slope 0.955). No flexible learner outperformed logistic regression; the paired AUROC difference with gradient boosting was 0.001 (95% CI −0.008 to 0.010). Within strata of previous diagnosis, discrimination was only 0.621 to 0.643. Model B provided net benefit above screen-all and screen-none strategies at thresholds above approximately 0.25, but reaching 80% sensitivity required flagging 66% of participants. **Conclusions:** Low-cost information can identify concurrently elevated gastrointestinal symptom burden with moderate accuracy. A parsimonious, well-calibrated logistic regression model performed comparably to more complex methods and is a reasonable candidate for community-based identification of current burden, not for forecasting risk. Prospective external validation and age-specific thresholds are required before clinical implementation.

## 1. Introduction

Symptoms arising from the gastrointestinal tract are among the most common health complaints in general populations. The Rome Foundation Global Study, which surveyed more than 73,000 adults across 33 countries, found that over 40% of respondents met criteria for at least one disorder of gut–brain interaction, with women affected more often than men and with substantial impairment of quality of life [1]. Meta-analytic estimates place the global prevalence of irritable bowel syndrome between roughly 4% and 10% depending on the diagnostic criteria applied, while gastroesophageal reflux disease affects an estimated 8% to 14% of adults [2,3]. The Rome IV framework organizes these conditions by symptom pattern rather than structural pathology, distinguishing functional bowel disorders from gastroduodenal syndromes such as functional dyspepsia [4,5,6]. Population surveys applying these criteria in high-income settings consistently show that individual symptoms are far more prevalent than formal diagnoses. They also show that symptom overlap across anatomical regions is associated with greater severity, poorer quality of life and higher healthcare utilization [7,8].

The clinical significance of this symptom burden is disproportionate to the attention it receives in resource-constrained health systems. Digestive conditions account for a considerable share of years lived with disability worldwide [9]. In Latin America they coexist with a high background prevalence of *Helicobacter pylori* infection, estimated at close to 60% in regional syntheses and substantially higher than in North America or Western Europe [10,11]. In Honduras, as in much of rural Central America, the practical consequence is that the first and often only clinical contact for abdominal pain, heartburn or bloating occurs at a primary-care post without access to endoscopy, breath testing or specialist referral within a reasonable travel distance. Under these conditions, the operational question is not which specific disorder a person has, but which people warrant limited assessment resources first. The Declaration of Astana framed exactly this challenge, calling for primary health care that uses available evidence to direct scarce capacity toward those who benefit most [12].

Machine learning has been proposed as a route to such prioritization, but the evidence base is geographically narrow. A systematic scoping review of artificial intelligence applications evaluated in real-world low- and middle-income country health settings identified only ten qualifying studies, almost none of which addressed gastrointestinal health [13]. Recent gastrointestinal applications of tabular machine learning have come mainly from hospital or health-check cohorts in East and South Asia. They include models for *Helicobacter pylori* infection built from routine laboratory and anthropometric data [14,15], a SHAP-explained model for gastric intestinal metaplasia among endoscoped inpatients [16], and a lifestyle-based scoring system for gastroesophageal reflux disease [17]. Explainable artificial intelligence has also been reviewed for inflammatory bowel disease [18]. Most of these studies relied on a single random split, laboratory predictors or synthetic oversampling, and none addressed community symptom burden in Central America. This matters beyond representation. Algorithms trained in one population encode that population’s measurement practices, care-seeking patterns, and outcome proxies. Transporting them elsewhere can systematically misallocate care, as shown when a widely deployed risk algorithm was found to under-refer Black patients because it predicted healthcare expenditure rather than illness [19,20]. Models intended for Central American primary care therefore need to be developed, evaluated, and reported transparently within those populations, following current guidance for prediction models that use regression or machine learning methods [21].

A second consideration concerns the choice of modeling strategy. Systematic comparison of published prediction studies has found no consistent discrimination advantage for machine learning over logistic regression once validation procedures are handled properly [22], and the calibration of flexible learners is frequently poor and rarely reported [23]. Recent commentary has argued that data quality and dataset characteristics, rather than algorithm choice, usually limit performance on clinical tabular data [24]. Benchmarks also show that deep neural networks rarely outperform tree ensembles on medium-sized tabular datasets [25], although tabular foundation models are beginning to change this picture [26]. The contribution of the present work is therefore methodological and contextual rather than algorithmic. Because a screening tool is useful only if its predicted probabilities support a defensible triage decision, discrimination alone is an inadequate criterion; net benefit across clinically plausible decision thresholds provides the more relevant evaluation [27]. Where flexible models are used, model-agnostic attribution methods such as SHAP allow the contribution of each variable to be inspected, provided attributions are interpreted as descriptions of model behavior rather than causal effects [28].

To our knowledge, no published study has developed and internally validated an explainable classification model for gastrointestinal symptom burden in a Central American community population. We asked whether explainable machine learning models built from low-cost demographic, behavioral, and lifestyle information can identify individuals with concurrently elevated gastrointestinal symptom burden in Honduran community settings, and whether these models offer plausible value for primary-care prioritization in a resource-constrained health system. The intended use population is narrow: community residents aged 12 to 49 years without a known diagnosis of structural or organic gastrointestinal disease. The findings do not extend to older adults or to patients presenting with alarm features. We hypothesized that a parsimonious model using information obtainable during a brief household interview would achieve moderate discrimination, that adding a single item on previously diagnosed digestive disease would materially improve performance, and that flexible learners would offer little or no advantage over well-specified logistic regression.

The models were intended for prioritization for further assessment, not for diagnosing a specific gastrointestinal disorder, and not for forecasting future gastrointestinal risk.

## 2. Materials and Methods

### 2.1. Study Design and Setting

This cross-sectional, community-based observational study was conducted in Honduras between 18 September and 19 November 2025. Data were collected in 85 communities distributed across 10 municipalities and five departments (Colón, Comayagua, El Paraíso, Intibucá and Olancho), covering coastal, central highland and interior settings with differing socioeconomic profiles. Reporting follows the STROBE recommendations for cross-sectional studies [29] and, for the prediction-model component, the TRIPOD+AI statement [21]. Analytical decisions and potential sources of bias were appraised against PROBAST+AI principles [30]. Items not applicable to this design were TRIPOD+AI items on treatments received, model updating and comparison of development and external validation data, and the STROBE items on follow-up and loss to follow-up. Items only partly addressed were blinding of outcome and predictor assessment (a single self-report instrument), the number of individuals approached and declining participation, and prospective registration. All items are mapped in Supplementary Tables S6 and S7.

Because predictors and outcome were measured at the same interview, the models estimate the probability that elevated symptom burden is present at the time of assessment. In TRIPOD terminology they are diagnostic-type (concurrent) classification models rather than prognostic models. The terms “prediction” and “risk” are used in this sense only and do not imply forecasting of future gastrointestinal events.

### 2.2. Study Population and Participant Selection

Eligible participants were residents aged 12 to 49 years who had lived in the community for at least six months and who provided informed consent, with parental or guardian consent and participant assent for those under 18 years. We applied two exclusion criteria at recruitment. The first excluded individuals with a previously established diagnosis of structural or organic gastrointestinal disease, that is, a condition confirmed by endoscopy, imaging, histology or an equivalent objective procedure, such as inflammatory bowel disease, celiac disease, peptic ulcer disease or gastrointestinal malignancy. This was because the study was directed at symptom burden in the absence of demonstrable organic pathology. The second excluded individuals who had received treatment for a gastrointestinal infection within the previous three months. Neither criterion excluded participants who had at some point been told by a clinician that they had a symptom-defined or infection-related digestive condition without objective confirmation. The questionnaire item on previous digestive diagnosis records exactly that broader, participant-reported category. The 399 affirmative responses to that item (21.7%) are therefore consistent with the eligibility criteria as applied, and the variable is admissible as a predictor in Model B. Because of the sampling design, all estimates describe the sampled communities and must not be interpreted as nationally representative.

Recruitment was organized around the catchment areas of 15 public primary-care facilities (Centros Integrales de Salud, Unidades de Atención Primaria en Salud and related units) that had agreements with the university for community health activities. Within each catchment area, interviewers approached residents in their homes or at the local health post over 26 fieldwork days and invited those who met the eligibility criteria. This was a convenience sample; communities and households were not randomly selected. The number of participants per community ranged from 1 to 142 (median 4, interquartile range 1 to 26), and 46 communities contributed five or fewer participants. The questionnaire did not record a household identifier, so the number of participants sharing a household cannot be determined. This clustering was addressed analytically through community-grouped validation and community-level resampling (Section 2.9).

Households were selected according to their geographical proximity to participating healthcare facilities, guided by existing household registries and geographic maps maintained by these institutions. Of the 460 households initially identified, 43 (9.3%) were excluded because the dwellings were either closed at the time of the field visit or uninhabited. The remaining 417 households (90.7%) constituted the recruitment pool, with no refusals reported among those successfully contacted. No other household-level exclusion criteria were applied.

### 2.3. Data Collection and Questionnaire

A structured, interviewer-administered questionnaire was applied face-to-face in the participant’s home or local health post. The instrument recorded sociodemographic characteristics, four current gastrointestinal symptom items, dietary and lifestyle behaviors, self-rated digestive health, and self-reported previous digestive diagnoses, both as a single global item and as a checklist of specific conditions (irritable bowel syndrome, gastritis, gastroesophageal reflux disease, colitis, *Helicobacter pylori* infection, other). Administration took approximately 10 min. The questionnaire did not record when a previous diagnosis had been made, its source, or whether current symptoms had begun before or after it.

The questionnaire records individual symptoms and does not implement the full Rome IV algorithm, which additionally requires symptom frequency thresholds, minimum duration, and relationship to defecation. No outcome in this study should therefore be read as a Rome IV diagnosis, nor as a diagnosis of irritable bowel syndrome, functional dyspepsia, or any other specific disorder of gut–brain interaction.

### 2.4. Outcome Definition

The prespecified primary outcome, elevated multi-symptom gastrointestinal burden, was defined as the presence of at least two of four current indicators: abdominal pain during the previous 30 days; heartburn during the previous two weeks; any reported gastroesophageal reflux above the response “never”; and post-prandial abdominal bloating.

This composite is a descriptive symptom-burden phenotype, not a validated clinical diagnosis. The threshold of at least two concurrent symptom indicators was prespecified to capture multisymptom gastrointestinal burden rather than isolated symptoms, while retaining sufficient outcome prevalence for classification modeling. Three considerations supported this threshold. First, single symptoms such as occasional reflux are very common in community surveys and are often transient, so a one-symptom definition would label most of the population. Second, population studies applying Rome IV criteria show that symptoms overlapping more than one anatomical region are associated with greater severity, somatization, impaired quality of life and higher healthcare use [8]. Third, co-occurrence of upper and lower abdominal symptoms is the pattern that most often prompts a primary-care consultation. To test whether conclusions depended on this choice, each symptom indicator and the 0-to-4 symptom count were also analyzed separately (Section 2.11).

Three prespecified alternative definitions were examined in sensitivity analyses: at least three of the four indicators, a stricter reflux definition counting only “frequently” or “always” as positive, and self-rated digestive health below “good” (that is, regular, poor or very poor).

### 2.5. Candidate Predictors

Predictors were restricted to information obtainable without laboratory investigation or specialist testing. Model A, the community screening model, comprised eight items: age in years, sex, educational attainment (coded as an ordinal scale from no formal schooling to completed tertiary education), daily consumption of fiber-rich foods, consumption of at least eight glasses of water per day, physical activity at least three times weekly, current cigarette smoking, and habitual alcohol consumption. Model B, the clinically augmented model, added a single item indicating a previously diagnosed digestive disorder.

Model A represents a purely community-based screening scenario using information obtainable without prior clinical history, whereas Model B represents a clinically augmented scenario in which self-reported previous digestive history is additionally available.

The four symptom items constituting the outcome were excluded from all predictor sets, as was self-rated digestive health, which is a subjective global rating of the same symptom experience and would therefore constitute outcome leakage. Previous digestive diagnosis was treated as a historical self-reported predictor rather than as a component of the current symptom-burden outcome. Its incremental contribution was therefore evaluated explicitly by comparing Models A and B. Because the diagnosis may itself reflect earlier episodes of the same symptoms or previous contact with health services, performance was additionally examined separately in participants with and without a previous diagnosis (Section 2.11).

Geography was used for subgroup and grouped-validation purposes only and was not included in either clinical model, because department membership in a convenience sample reflects the field-work allocation rather than a transportable patient characteristic. With 840 events and a maximum of nine estimated parameters, the events-per-parameter ratio exceeded 90, comfortably above conventional requirements for stable model estimation [31].

### 2.6. Data Quality and Preprocessing

The delivered dataset contained 1838 records and 33 variables with no missing cells. All ages fell within the eligible 12-to-49-year range, and all binary items were coded 0 or 1. A complete audit was performed before any modelling, and every recoding rule was logged (Supplementary Table S1).

Categorical labels were normalized for leading, trailing and repeated whitespace, which collapsed variant spellings of one municipality and one health facility. Seven records carried a health-region code corresponding to a department in which no sampled community was located, while their department, municipality, community and health facility were mutually consistent. The region code was harmonized to the department-consistent value, and the original field was retained unmodified. Two records carried collection dates outside the principal fieldwork window. Dates were not overwritten; the variable was excluded from modelling and a sensitivity analysis excluding those records is reported. Seventeen records, forming eight groups, were identical across all 31 substantive fields. Since the instrument is anonymous, indistinguishable records cannot be shown to be duplicate data entries rather than distinct respondents with identical response profiles. All were therefore retained for the primary analysis, with a sensitivity analysis removing the redundant copies.

The global item on previous digestive diagnosis and the checklist of specific diagnoses disagreed in 23 records. A harmonized variable was defined as an affirmative response to either source; both original fields were retained, and a sensitivity analysis using the unharmonized global item alone is reported. Harmonization was chosen because the two items describe the same construct and each can fail independently. Eleven participants denied a previous diagnosis on the global item but then ticked a specific condition, which is more plausibly a skipped global item than a false checklist entry. The remaining 12 answered yes globally without ticking a condition, and five of them described it in free text. Treating either source as sufficient therefore minimizes misclassification of the predictor.

Administrative fields describing the data-collection structure, the free-text name of the individual data collector, and the name of the health facility were removed from the analytical dataset before modelling. Facility name was excluded from the candidate predictor pool because it encodes collection logistics rather than a clinically meaningful exposure.

### 2.7. Descriptive Statistical Analysis

Continuous variables are summarized as means with standard deviations and categorical variables as counts with percentages. Proportions are reported with 95% Wilson confidence intervals. Comparisons between outcome groups used the Welch *t* test for continuous variables and the chi-square test for categorical variables, with effect sizes reported as Cohen’s d, odds ratios or Cramer’s V alongside confidence intervals, so that interpretation did not rest on *p* values alone. The intraclass correlation of the outcome within communities was estimated with the one-way analysis-of-variance estimator, together with the corresponding design effect.

### 2.8. Machine Learning Model Development

The task was binary classification: each model estimated the probability that a participant had elevated symptom burden at the time of the interview. Logistic regression was therefore used as a probabilistic classifier for a binary outcome, not as a regression model for a continuous response. It also served as the conventional reference model and as the source of adjusted odds ratios. The four principal algorithms were compared under identical evaluation conditions: multivariable logistic regression as the conventional reference, elastic-net penalized logistic regression, random forest [32], and gradient-boosted trees implemented in XGBoost 3.4.1 [33]. Three further classifiers were added during revision as comparators: a single decision tree, a support vector machine with radial basis kernel and Platt-scaled probabilities, and a feed-forward artificial neural network (multilayer perceptron with one or two hidden layers, L2 regularization and early stopping). Deep architectures were not pursued because tree ensembles and regression generally match or outperform them on medium-sized tabular data with few predictors [25]. Continuous predictors were standardized within each training fold for the regression, support vector machine and neural network models. No synthetic oversampling was applied, since the outcome prevalence of 45.7% presents no meaningful class imbalance; class weighting was likewise unnecessary.

### 2.9. Internal Validation and Hyperparameter Optimization

Models were evaluated by nested cross-validation. The outer loop used five-fold stratified cross-validation repeated five times, yielding 25 development-evaluation cycles and out-of-fold predictions for every participant in each repetition. In each cycle, 80% of participants (approximately 1470) formed the development set and 20% (approximately 368) the evaluation set, so every participant was used for evaluation exactly once per repetition. Repetition with different random partitions was used to quantify the variability attributable to the particular split, not to enlarge the sample. Within each outer training set, an inner three-fold stratified cross-validation (two thirds for fitting and one third for scoring) performed the hyperparameter grid search, optimizing the area under the receiver operating characteristic curve. Classification thresholds were also derived exclusively within the outer training folds from inner cross-validated predictions using the Youden index, and were then applied unchanged to the held-out fold. No hyperparameter or threshold was selected using data from an evaluation fold, and no model was fitted to the complete dataset before evaluation. Search spaces and the configurations selected most frequently are reported in Supplementary Table S2, and performance by repetition and by fold in Supplementary Table S3.

To examine sensitivity to the number of folds and possible overfitting, the complete nested procedure was repeated with ten-fold outer cross-validation (90% development and 10% evaluation per cycle), repeated five times for all algorithms except random forest, which was repeated three times because of computational cost. For each algorithm we also compared the apparent (training-fold) AUROC with the held-out AUROC; the difference is an estimate of optimism.

Because participants were recruited in geographic clusters, a second validation strategy repeated the entire nested procedure using StratifiedGroupKFold with community as the grouping variable, so that no community contributed observations to both development and evaluation folds. All validation reported here is internal; none of it constitutes external validation.

### 2.10. Model Performance and Calibration

For each model we report the area under the receiver operating characteristic curve, the area under the precision–recall curve, sensitivity, specificity, positive and negative predictive values, F1 score, balanced accuracy, Brier score, and calibration intercept and slope. Calibration intercept and slope were estimated by generalized linear models of the outcome on the linear predictor, with the intercept obtained using the linear predictor as an offset [23]. Calibration was displayed graphically using decile bins with Wilson intervals [34]. Confidence intervals were obtained by percentile bootstrap with 2000 resamples applied to the out-of-fold predictions averaged across repetitions; variability across repetitions is reported separately as the standard deviation of the AUROC. Fold-level variability is reported as the mean, standard deviation and t-based 95% confidence interval of the AUROC across the 25 (or 50) held-out folds. Because folds share training data, these fold-level intervals are descriptive.

Because participants were clustered in communities, all confidence intervals for the principal models were recomputed with a community-level cluster bootstrap. In this procedure the 85 communities, rather than individuals, were resampled with replacement 2000 times [35]. Differences between models were estimated as paired differences on the same bootstrap resamples, at both participant and community level. The differences covered AUROC, AUPRC, Brier score and net benefit at threshold probabilities of 0.30, 0.40 and 0.50. The paired AUROC difference was also tested with the DeLong method [36].

The Youden index was retained as a neutral reference threshold that weights sensitivity and specificity equally. It is not, by itself, a clinical decision rule [37]. Three clinically anchored alternatives were therefore examined. The first was a sensitivity-anchored threshold, chosen within each outer training fold as the highest threshold that achieved at least 80% sensitivity in the inner cross-validated predictions and then applied unchanged to the held-out fold. This threshold reflects a priority on not missing symptomatic individuals. The second was a set of capacity-anchored thresholds, defined by the proportion of the population that a team could assess (20% to 70%). The third was a set of harm-ratio thresholds from 0.25 to 0.50. A threshold probability p_t_ implies that one missed symptomatic person is weighed as (1 − p_t_)/p_t_ unnecessary assessments, so 0.30 and 0.50 correspond to ratios of about 2.3:1 and 1:1 [38]. Because Youden thresholds differ slightly between algorithms, threshold-dependent metrics were also compared at a common threshold probability of 0.40.

### 2.11. Explainability, Secondary Endpoints and Subgroup Evaluation

Adjusted odds ratios with 95% confidence intervals from the multivariable logistic model provide epidemiologically interpretable effect estimates; community cluster-robust confidence intervals are also reported. The logistic model is displayed as a forest plot of adjusted odds ratios. Its participant-level contributions are shown as exact linear SHAP values, each equal to the coefficient multiplied by the centered predictor value, so the regression and the tree ensemble can be compared on the same scale. The full model equation is given in Supplementary Table S9. For the gradient-boosting model, SHAP values were computed with the tree-specific exact algorithm [28,39], and global importance was summarized as the mean absolute SHAP value in log-odds units. The direction of each variable’s contribution was quantified by the Spearman correlation between feature value and its SHAP attribution. SHAP values describe the behavior of a fitted model and are not causal effect estimates.

As secondary endpoints, Models A and B were refitted with logistic regression for each of the four symptom indicators separately and for the strict reflux definition, using five-fold cross-validation repeated five times. The symptom count (0 to 4) was analyzed with a proportional-odds ordinal logistic model.

Performance was evaluated within subgroups defined by sex, age category (12 to 17, 18 to 29, 30 to 39 and 40 to 49 years), department, previous digestive diagnosis, and two proxies for access to care, reporting AUROC with bootstrap confidence intervals, sensitivity, specificity, positive predictive value, Brier score and calibration. The access proxies were educational attainment (primary schooling or less versus secondary or higher) and type of health facility serving the community (Centro Integral de Salud versus other primary-care units). Within these subgroups the paired gain in AUROC from Model A to Model B was estimated with bootstrap confidence intervals. Effect modification of the previous-diagnosis association was tested with interaction terms. Subgroups with fewer than 10 events or 10 non-events were not analyzed, and estimates from small strata are interpreted with caution. Two strategies for subgroup performance were then tested within the nested procedure. The first was age-specific thresholds (Youden or 80% sensitivity), derived in the training folds for each age band. The second was recalibration-in-the-large, in which the model intercept was re-estimated within each age band or department from training-fold predictions [40].

### 2.12. Decision Curve Analysis

Net benefit was calculated across threshold probabilities from 0.05 to 0.70 and compared with the strategies of prioritizing no participant and prioritizing all participants [27,41]. The threshold probability is interpreted here as the point at which a primary-care team would consider a structured digestive assessment worthwhile given the relative costs of unnecessary assessment and missed symptomatic individuals. To express net benefit in operational terms, we also report the net reduction in assessments per 100 people compared with assessing everyone [38].

### 2.13. Statistical Software and Reproducibility

The analysis code was written in Python by the study team. All analyses reported in this revision were re-executed in Python 3.13 using pandas 3.0.5, NumPy 2.5.3, SciPy 1.18.1, statsmodels 0.15.0, scikit-learn 1.9.1 [42], XGBoost 3.4.1, SHAP 0.53.0 and Matplotlib 3.11.2. Computation ran on a CPU-only Linux workstation (Intel Xeon processor, 2.1 GHz, two cores, 8 GB RAM); no graphics processing unit was used. The complete nested pipeline for all seven algorithms required about 27 min. A single random seed, 20,251,212, governed all stochastic components. The complete analysis script reproduces every reported value, table and figure from the source spreadsheet and is provided as Supplementary Material.

### 2.14. Ethical Considerations

The study protocol was reviewed and approved without reservations, by unanimous decision of a full quorum, by the Comité de Ética en Investigación Científica en Salud (CEICS) of the Colegio de Nutricionistas y Dietistas de Honduras (approval number CEICS-cndh 002-10-2025, March 2025, valid for 12 months and renewable subject to six-monthly progress reporting). The committee assessed the protocol against the Declaration of Helsinki [43], the CIOMS International Ethical Guidelines for Health-related Research Involving Humans (2016) with particular reference to vulnerable populations, the United Nations Convention on the Rights of the Child, and Pan American Health Organization guidance for community health research. It classified the study as low risk on the grounds that it involved a non-invasive questionnaire.

Written informed consent was obtained from all adult participants. For participants younger than 18 years, written consent was obtained from a parent or guardian together with written assent from the participant. In communities with low literacy, consent was obtained in writing or, where a participant could not read the form, before a witness, in accordance with a condition of the approval. Participation was voluntary, could be withdrawn at any time without consequence, and was not compensated. Responses were recorded under a numeric code without personal identifiers, and free-text fields identifying data collectors were removed before analysis. Data were held on secure institutional servers as required by the approval conditions.

## 3. Results

### 3.1. Participant Characteristics

**Figure 1:**
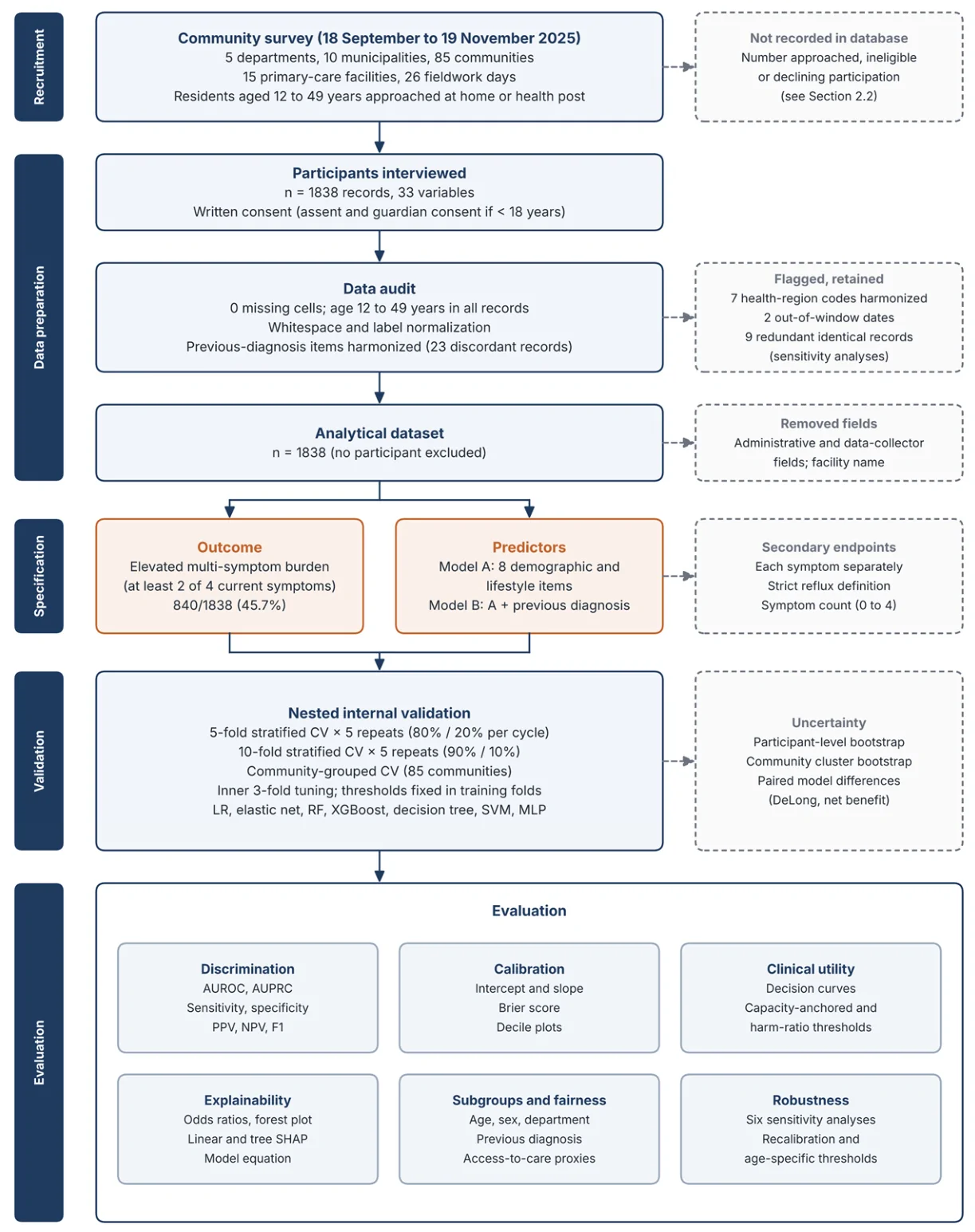
Study and analytical workflow. Flow of records from the source community survey through the data audit, removal of administrative and collector-identifying variables, definition of the outcome and predictor sets, nested internal validation, and evaluation components. All 1838 records met eligibility criteria and had complete data on analyzed variables; no participant was excluded from the primary analysis. The number of residents approached, found ineligible or declining participation was not captured in the analytical database (see Section 2.2).

The analytical dataset comprised 1838 participants with complete data on all analyzed variables (Figure 1). They were recruited from 85 communities served by 15 primary-care facilities; the median number of participants per community was 4 (range 1 to 142). Mean age was 28.3 years (SD 10.9), and 1198 participants (65.2%) were women. Educational attainment was concentrated in the primary and incomplete-secondary categories, and 42 participants (2.3%) reported no formal schooling. Participant characteristics according to outcome status are shown in Table 1.

Participants with elevated symptom burden were on average four years older (Cohen’s d 0.39, 95% CI 0.30 to 0.49) and reported markedly more adverse behavioral profiles. Current smoking was three times more frequent (16.2% versus 5.3%; OR 3.44, 95% CI 2.47 to 4.80) and habitual alcohol consumption almost three times more frequent (23.8% versus 10.0%; OR 2.81, 95% CI 2.16 to 3.64). Regular physical activity, daily fiber intake and adequate water intake were all less common. Sex was not associated with the outcome (OR 1.05, 95% CI 0.87 to 1.28). The strongest single association was a previous diagnosis of digestive disease, reported by 36.9% of those with elevated burden and 8.9% of those without (OR 5.97, 95% CI 4.61 to 7.74). The intraclass correlation of the outcome within communities was 0.065. Because a few communities contributed many participants, this corresponds to a design effect of 5.59 for the estimation of prevalence, so the participant-level Wilson intervals in Table 2 understate uncertainty about community prevalence.

### 3.2. Gastrointestinal Symptom Burden

Reflux above the “never” category was the most frequently reported indicator, affecting 1017 participants (55.3%, 95% CI 53.1 to 57.6), although only 195 (10.6%) described it as frequent or constant. Abdominal pain, heartburn and post-prandial bloating each affected approximately one third of the sample (Table 2). Symptoms clustered rather than occurring in isolation: 543 participants (29.5%) reported none of the four indicators, 455 (24.8%) reported one, and 453 (24.6%) reported three or four.

The prespecified primary outcome, at least two of four indicators, was present in 840 participants (45.7%, 95% CI 43.4 to 48.0). Self-rated digestive health tracked this composite closely: 39.3% of all participants rated their digestive health below “good”. The association between the two was the strongest observed in the descriptive analysis (Cramer’s V 0.503). This supports the face validity of the composite while also explaining why the self-rating was excluded from the predictor set. Self-reported previous digestive diagnoses were dominated by *Helicobacter pylori* infection (7.6%) and gastritis (7.4%), with irritable bowel syndrome reported by only 47 participants (2.6%).

Among the 840 participants with elevated burden, 453 (53.9%) reported three or four indicators and 182 (21.7%) all four. In addition, 63.6% rated their digestive health as fair or worse, 15.1% as poor or very poor, and 36.9% reported a previous digestive diagnosis. Two thirds (66.5%) reported heartburn or frequent reflux, the symptom pattern for which empirical acid suppression or *Helicobacter pylori* test-and-treat is recommended in primary care [44].

### 3.3. Model Development and Performance

Using demographic and lifestyle information alone, all four principal algorithms achieved similar and modest discrimination, with an AUROC between 0.660 and 0.663 (Table 3). Adding the single item on previously diagnosed digestive disease raised discrimination to between 0.723 and 0.726, an increase of approximately 0.06 in AUROC that was consistent across algorithms and across repetitions.

**Figure 2:**
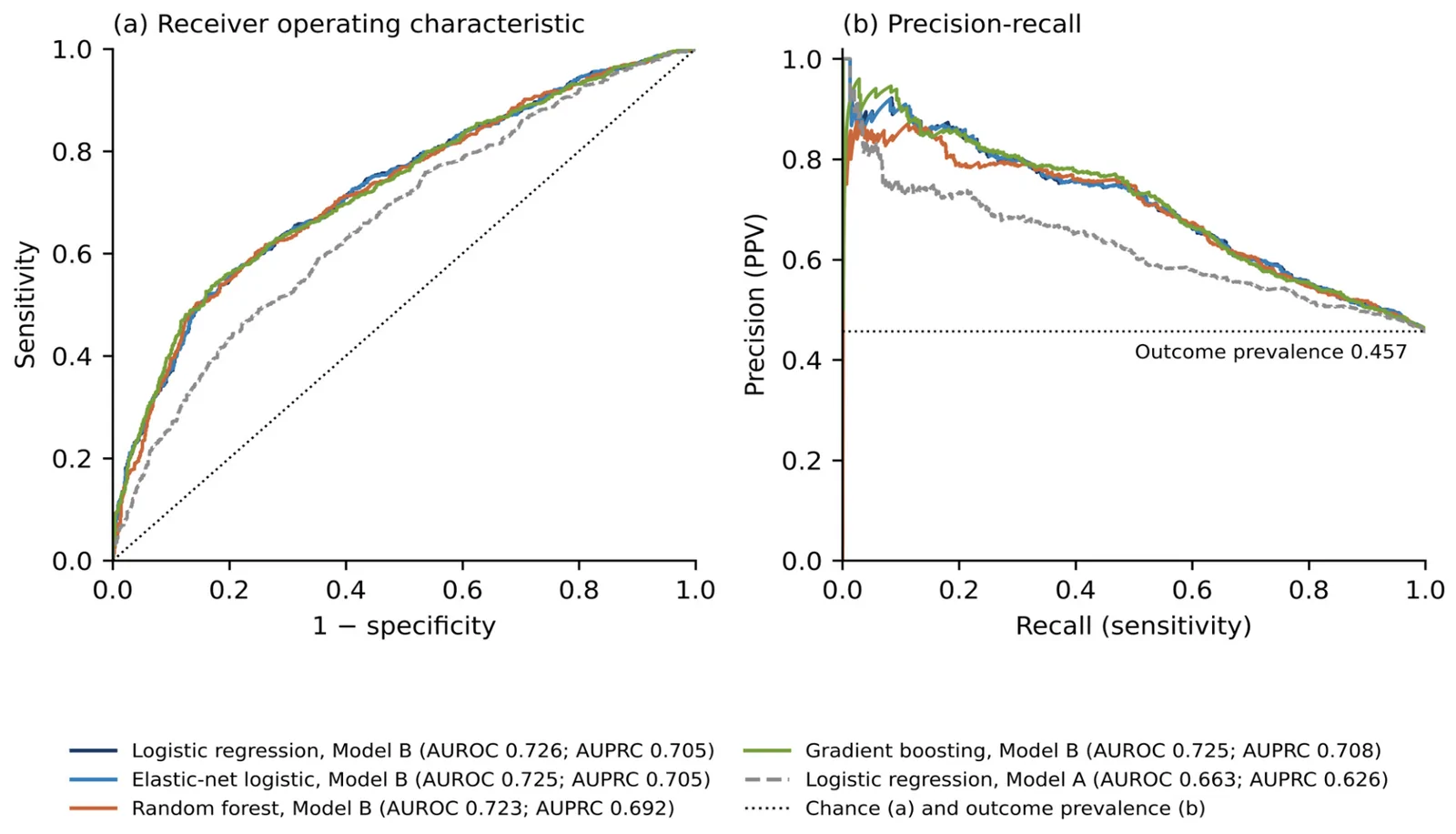
Discrimination of candidate classification models. (**a**) Receiver operating characteristic curves and (**b**) precision–recall curves for the four principal Model B algorithms, with Model A logistic regression shown for comparison. Curves are derived from out-of-fold predictions averaged across five repetitions of stratified five-fold nested cross-validation (*n* = 1838 840 events). The dotted diagonal in panel (**a**) denotes chance performance; the dotted horizontal line in panel (**b**) denotes the outcome prevalence of 0.457, which is the expected precision of a non-informative classifier. AUROC, area under the receiver operating characteristic curve; AUPRC, area under the precision–recall curve; PPV, positive predictive value.

Logistic regression showed performance comparable to the more flexible machine-learning approaches in both feature sets. In Model B, it achieved an AUROC of 0.726 (95% CI 0.702 to 0.748) and an AUPRC of 0.705 (95% CI 0.674 to 0.737) against a background prevalence of 0.457. Gradient boosting was almost identical (AUROC 0.725, 95% CI 0.700 to 0.748; AUPRC 0.708), and random forest was marginally lower (0.723). With community-level resampling, the interval for the logistic AUROC widened to 0.675 to 0.772. Paired bootstrap differences confirmed that none of these differences was material. The AUROC difference between logistic regression and gradient boosting was 0.001 (95% CI −0.008 to 0.010; DeLong *p* = 0.87). The corresponding differences in AUPRC (−0.003, 95% CI −0.014 to 0.007), Brier score (0.0010, 95% CI −0.0016 to 0.0036) and net benefit at a threshold of 0.40 (0.007, 95% CI −0.005 to 0.019) all had confidence intervals spanning zero. Against elastic net the AUROC difference was only 0.0005. The DeLong test nevertheless gave *p* = 0.036, because the two sets of predictions were almost perfectly correlated; a difference of this size has no practical relevance. The three additional classifiers did not improve on logistic regression: the support vector machine reached an AUROC of 0.720 (95% CI 0.697 to 0.743), the neural network 0.713 (95% CI 0.689 to 0.737) and the single decision tree 0.704 (95% CI 0.679 to 0.728). Variability across the five repetitions was negligible for every model (AUROC standard deviation 0.002 to 0.007). Across the 25 held-out folds, the Model B logistic AUROC averaged 0.726 (standard deviation 0.026; 95% CI across folds 0.715 to 0.736; Supplementary Table S10). At the Youden threshold fixed within the training folds (0.475), Model B logistic regression achieved 55.5% sensitivity, 79.8% specificity, 69.8% positive predictive value and 68.0% negative predictive value. Discrimination is displayed in Figure 2.

Community-grouped cross-validation, in which no community appeared in both development and evaluation folds, produced almost no degradation for the regression models (Model B logistic regression AUROC 0.722, 95% CI 0.698 to 0.745) but a larger decline for the tree ensembles (random forest 0.711; gradient boosting 0.712). The corresponding Model A estimates were 0.656 and 0.640. This pattern suggests that the flexible learners were absorbing a small amount of community-level structure that does not transport across geographic clusters. Resampling whole communities approximately doubled the width of the confidence interval for the Model B logistic AUROC, to 0.675 to 0.772 (participant-level 0.702 to 0.748). This reflects heterogeneity between communities. Paired differences between algorithms were much less affected, because both models are evaluated on the same resampled communities, and no comparison changed (Table 4; Supplementary Table S13).

Ten-fold nested cross-validation gave results that were practically identical to five-fold validation (Supplementary Table S10). The Model B logistic AUROC was 0.726 with ten folds, against 0.726 with five; across all seven algorithms the two schemes differed by at most 0.004. The apparent (training-fold) AUROC exceeded the held-out AUROC by only 0.008 for logistic regression and 0.006 for the strongly regularized support vector machine. The gap was 0.018 for the neural network, 0.048 for random forest and 0.049 for gradient boosting. The tree ensembles therefore fitted the training folds more closely without any gain on held-out data, whereas logistic regression showed minimal optimism.

Since a screening application generally tolerates lower specificity, we also examined a higher-sensitivity operating point. The 80% sensitivity threshold derived within the training folds averaged 0.326 (range across folds 0.314 to 0.341). This is the basis of the value of 0.327 reported in the original submission, which had been located on the pooled out-of-fold predictions. Applied to the held-out predictions, the nested threshold yielded 80.0% sensitivity, 45.3% specificity and 55.2% positive predictive value while flagging 66.3% of the sample. This illustrates the limited efficiency gain that a model of this discrimination can deliver at high sensitivity when the underlying condition affects nearly half the population.

Youden thresholds differed slightly between algorithms (0.473 to 0.503), so the threshold-dependent metrics in Table 3 partly reflect where each threshold fell rather than differences in ranking ability. At a common threshold probability of 0.40, sensitivity ranged from 65.1% to 70.1% and specificity from 61.7% to 68.3% across the four principal Model B algorithms. The ordering of models was the same as for the threshold-free AUROC and Brier score.

### 3.4. Calibration and Clinical Utility

**Figure 3:**
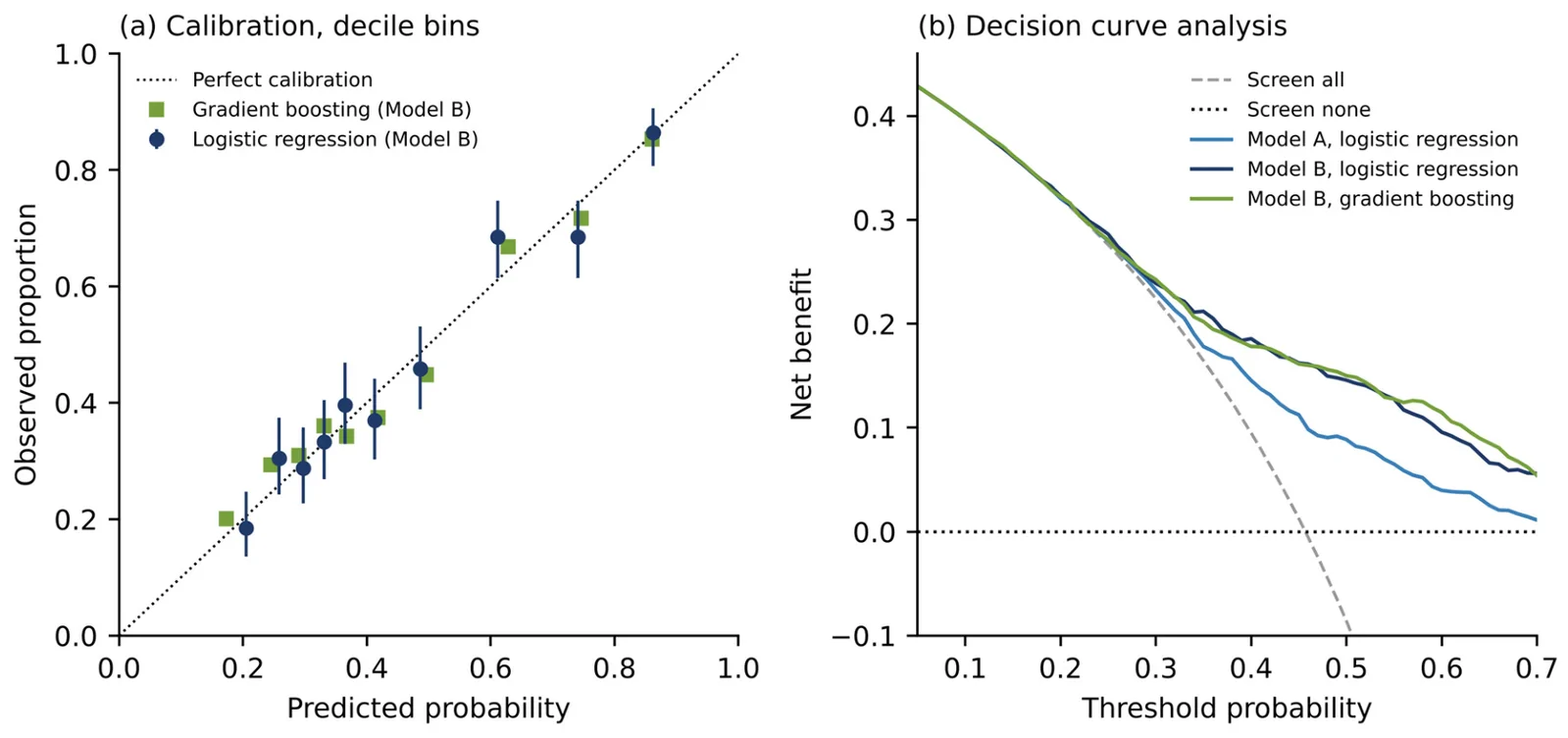
Calibration and clinical utility. (**a**) Calibration plot showing observed proportions against mean predicted probabilities within deciles of predicted risk for Model B logistic regression (circles, with 95% Wilson intervals) and Model B gradient boosting (squares); the dotted line indicates perfect calibration. (**b**) Decision curve analysis showing net benefit across threshold probabilities from 0.05 to 0.70 for Model A logistic regression, Model B logistic regression and Model B gradient boosting, compared with the strategies of screening all participants (dashed) and screening none (dotted). All predictions are out-of-fold (*n* = 1838).

Model B logistic regression was well calibrated across the full range of predicted risk, with a calibration intercept of 0.000 and a calibration slope of 0.955 (95% CI 0.838 to 1.081), and a Brier score of 0.208 (95% CI 0.200 to 0.216). Gradient boosting was slightly over-fitted in the same sense, with a calibration slope of 0.930 and a marginally better Brier score of 0.206. Random forest was clearly miscalibrated, with a calibration slope of 1.254 (95% CI 1.104 to 1.420) reflecting predictions compressed toward the sample prevalence. Among the added classifiers, the calibration slope was 0.917 for the support vector machine, 1.052 for the neural network and 0.927 for the decision tree. Decile-binned observed proportions for the two best models are shown in Figure 3a.

Decision curve analysis (Figure 3b) showed that both Model B strategies produced greater net benefit than prioritizing all participants at threshold probabilities above approximately 0.25, and greater net benefit than prioritizing none across the entire range examined. At a threshold probability of 0.40, net benefit was 0.186 for Model B logistic regression compared with 0.095 for prioritizing everyone; at 0.50 the corresponding values were 0.146 and negative. Model A produced consistently lower net benefit than Model B above a threshold of about 0.30. In paired comparisons, the net-benefit gain of Model B over Model A was 0.040 (95% CI 0.022 to 0.057) at a threshold of 0.40 and 0.057 (95% CI 0.035 to 0.079) at 0.50, but only 0.007 (95% CI −0.005 to 0.018) at 0.30 (Table 4). Below a threshold of approximately 0.20, no model improved on simply assessing everyone, which is the expected consequence of a condition affecting close to half the population.

Expressed operationally (Table 5), the gain was small at the thresholds a screening program would favor. At a threshold of 0.30, the model flagged 74.3% of participants, detected 85.4% of those with elevated burden and avoided a net 3.4 assessments per 100 people relative to assessing everyone. At the nested 80% sensitivity threshold it avoided a net 5.7 assessments per 100 and missed about 9 symptomatic people per 100 screened. Material efficiency gains appeared only at thresholds of 0.40 or above. At 0.40 the model flagged 47.2% of participants and avoided a net 13.6 assessments per 100, but sensitivity fell to 65.7%. When capacity, rather than a probability threshold, was the constraint, assessing the 30% at highest predicted risk captured 48.9% of affected individuals with a positive predictive value of 74.5%.

### 3.5. Model Explainability

**Figure 4:**
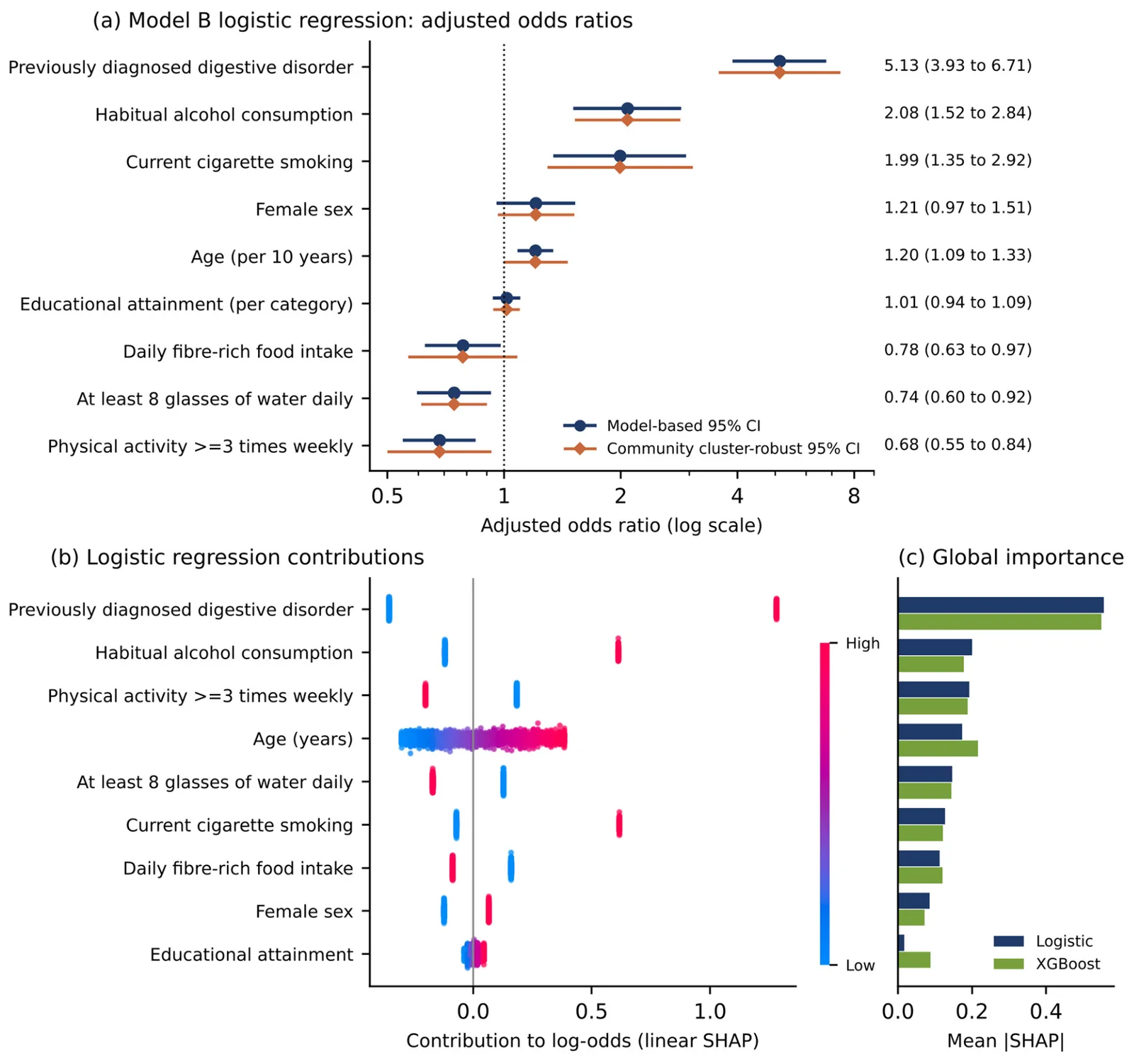
Visual summary of the Model B logistic regression. (**a**) Adjusted odds ratios with model-based 95% confidence intervals (circles) and community cluster-robust 95% confidence intervals (diamonds), on a logarithmic scale; numbers give the odds ratio and model-based interval. (**b**) Participant-level contributions to the log-odds (exact linear SHAP values, coefficient multiplied by centered predictor value), colored by predictor value (blue low, red high). (**c**) Global importance (mean absolute SHAP value) for logistic regression and gradient boosting on the same log-odds scale. The model was fitted to all 1838 participants for display; performance estimates come from nested cross-validation. Associations are cross-sectional and not causal.

Adjusted odds ratios from the multivariable logistic model are reported in Table 6 and displayed in Figure 4a. After mutual adjustment, previously diagnosed digestive disease remained by far the strongest correlate of elevated symptom burden (OR 5.13, 95% CI 3.93 to 6.71), followed by habitual alcohol consumption (OR 2.08, 95% CI 1.52 to 2.84) and current smoking (OR 1.99, 95% CI 1.35 to 2.92). Each additional decade of age was associated with 20% higher odds (OR 1.20, 95% CI 1.09 to 1.33). Regular physical activity (OR 0.68, 95% CI 0.55 to 0.84), adequate water intake (OR 0.74, 95% CI 0.60 to 0.92) and daily fiber intake (OR 0.78, 95% CI 0.63 to 0.97) were each associated with lower odds. Educational attainment showed no independent association, and the association with female sex was weak and not statistically significant after adjustment (OR 1.21, 95% CI 0.97 to 1.51). Community cluster-robust intervals were wider for age, fiber intake and physical activity (Table 6). The interval for daily fiber intake then included 1 (0.57 to 1.07), so this association should be regarded as uncertain; the other conclusions were unchanged.

Because logistic regression was the preferred model, its behavior is shown directly in Figure 4. The linear SHAP decomposition of the logistic model ranked previous diagnosis first (mean absolute contribution 0.556 log-odds units), followed by alcohol consumption (0.200), physical activity (0.192) and age (0.173). This ordering is almost the same as that of gradient boosting (Figure 4c). The main difference was educational attainment, to which the logistic model assigned almost no weight (0.016), against 0.088 in gradient boosting. The intercept and coefficients needed to compute an individual probability are reported in Supplementary Table S9.

**Figure 5:**
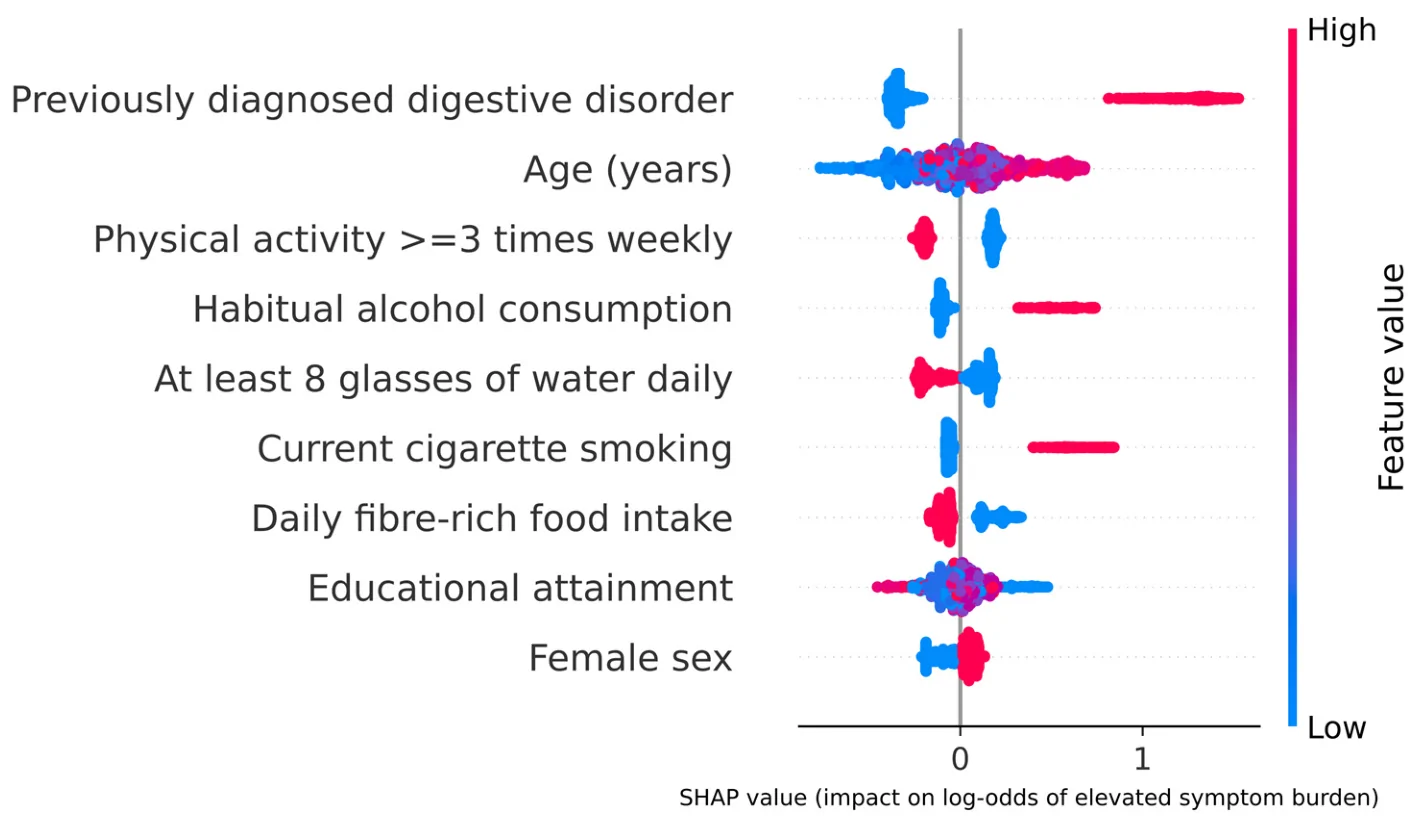
SHAP summary for the Model B gradient-boosting classifier. Each point represents one participant, positioned by the SHAP value of the indicated variable in log-odds units and colored by the value of that variable (blue low, red high). Variables are ordered by mean absolute SHAP value. Positive values indicate that the variable increased the model’s predicted log-odds of elevated gastrointestinal symptom burden for that participant. SHAP values describe the behavior of the fitted model and are not causal effect estimates. The model was fitted to the complete dataset using the hyperparameter configuration most frequently selected during nested cross-validation, and is used here for interpretation rather than performance estimation.

SHAP analysis of the gradient-boosting model produced a concordant ordering (Figure 5). Previous digestive diagnosis dominated global importance (mean absolute SHAP 0.549 log-odds units), followed by age (0.216), physical activity (0.189) and alcohol consumption (0.178). Water intake, smoking and fiber intake contributed comparably and more modestly (0.144, 0.122 and 0.121), while education (0.088) and sex (0.072) contributed least. Directions were consistent with the regression estimates: higher age, smoking, alcohol use and prior diagnosis pushed predictions upward, whereas physical activity, adequate hydration and fiber intake pushed them downward. These attributions describe how the fitted model uses the available variables and should not be read as causal effects.

### 3.6. Subgroup and Geographic Robustness Analyses

Performance varied across subgroups (Table 7). Discrimination was higher in men (AUROC 0.759, 95% CI 0.721 to 0.796) than in women (0.703, 95% CI 0.673 to 0.731), with overlapping but distinct confidence intervals. Performance was weakest in adolescents aged 12 to 17 years (AUROC 0.650, 95% CI 0.589 to 0.718), the subgroup with the lowest outcome prevalence (31.0%). In this group, the fixed threshold produced high specificity (93.3%) but sensitivity of only 31.7%. In the two oldest age bands, where prevalence exceeded 54%, the same threshold produced the opposite profile.

Across departments, the AUROC ranged from 0.665 in Colón and Olancho to 0.794 in Comayagua. The apparently high estimate in El Paraíso (0.848) rests on only 53 participants and a calibration slope of 2.07 and should not be interpreted as evidence of superior performance. Calibration was adequate in the larger departments but showed a systematic intercept shift in Intibucá, where predicted risks exceeded observed prevalence (calibration intercept minus 0.242), consistent with the lower observed burden in that department. These findings indicate that a single global operating threshold may not provide comparable screening performance across age groups and geographic settings.

The gain from adding previous diagnosis operated almost entirely between, rather than within, the two diagnosis strata (Table 8). The outcome was present in 77.7% of participants reporting a previous diagnosis and in 36.8% of those without one. Within each stratum, Model B discriminated no better than Model A: the AUROC was 0.643 (95% CI 0.611 to 0.674) among the 1439 participants without a previous diagnosis and 0.621 (95% CI 0.560 to 0.689) among the 399 with one. Model B therefore adds a strong signal for one identifiable group but does not improve ranking within it.

If the previous-diagnosis item mainly encoded access to care, its frequency and its association with the outcome would be expected to vary with markers of access. The proportion reporting a previous diagnosis was almost identical across educational groups (21.5% and 21.8%). It was higher in communities served by Centros Integrales de Salud (25.5%) than in those served by other primary-care units (14.4%). However, the adjusted odds ratio for previous diagnosis did not differ by education (interaction *p* = 0.79) or facility type (interaction *p* = 0.91). The gain in AUROC from Model A to Model B was similar across these groups (Table 8), ranging from 0.052 to 0.077.

Two strategies to improve subgroup performance were tested within the nested procedure (Table 5). Recalibration-in-the-large by age band changed the calibration intercept in adolescents from minus 0.076 to minus 0.002 but left sensitivity unchanged (31.7%). The low sensitivity therefore reflects lower predicted risks in a lower-prevalence group, not miscalibration. Age-specific Youden thresholds raised sensitivity in the 18-to-29-year band (from 45.5% to 51.3%) but not in adolescents, where the optimal Youden point remained a high-specificity point. Age-specific thresholds targeting 80% sensitivity equalized detection across age bands (79.3% to 81.7%), at the cost of specificity between 34.5% and 45.1%. Recalibration-in-the-large by department removed the intercept shifts in Intibucá (from minus 0.242 to 0.001) and Olancho (from 0.457 to 0.004), with an overall Brier score of 0.207.

Six sensitivity analyses (Supplementary Table S4) gave stable results. Across the six analyses, the AUROC ranged from 0.659 to 0.673 for Model A and from 0.718 to 0.745 for Model B logistic regression, compared with 0.663 and 0.726 in the primary analysis. Discrimination was slightly higher for the more restrictive endpoints (AUROC 0.741 for at least three of four symptoms and 0.745 for the strict reflux definition) and slightly lower for the self-rated digestive health endpoint (0.718). Excluding the nine redundant records, excluding the two records with out-of-window dates, and substituting the unharmonized prior-diagnosis item each changed the AUROC by 0.005 or less. With the unharmonized global item, the adjusted odds ratio for previous diagnosis was 5.20 (95% CI 3.96 to 6.83), against 5.13 for the harmonized variable. Harmonization therefore did not change any conclusion.

The individual symptoms and the symptom count gave the same picture as the composite outcome (Supplementary Table S8). With Model B, the AUROC was 0.677 for abdominal pain, 0.714 for heartburn, 0.690 for any reflux, 0.761 for frequent or constant reflux and 0.669 for bloating. Model A values ranged from 0.612 to 0.686. Previous diagnosis and alcohol consumption were associated with every individual symptom. Smoking was associated with every symptom except frequent or constant reflux (OR 1.30, 95% CI 0.81 to 2.09). In the ordinal model for the zero-to-four symptom count, the adjusted odds ratios were 5.22 (95% CI 4.21 to 6.47) for previous diagnosis, 2.01 for alcohol, 1.90 for smoking and 0.77 for physical activity. Mean predicted probability from the primary model rose steadily with symptom count, from 0.355 with no symptoms to 0.647 with all four (Spearman correlation 0.43). The AUROC increased from 0.693 for any symptom to 0.753 for all four. The two-symptom threshold therefore sits on a continuous gradient rather than creating an artificial signal.

## 4. Discussion

Nearly half of the community members surveyed in these Honduran localities reported at least two concurrent gastrointestinal symptoms, and inexpensive information collected in a ten-minute household interview identified those individuals with moderate accuracy. The central quantitative finding is a decomposition of where that accuracy comes from. Demographic and lifestyle information alone supported an AUROC of 0.663, which is too low to guide individual triage. A single additional question about previously diagnosed digestive disease raised discrimination to 0.726, contributed roughly half of the total model signal in the SHAP analysis, and carried an adjusted odds ratio above five. The practical implication is favorable for deployment: the marginal cost of that question in a household survey is essentially zero, and it does more work than the entire lifestyle battery combined. However, Model B’s incremental performance should be interpreted cautiously because prior diagnostic labeling may capture persistence of gastrointestinal symptoms, prior healthcare contact, or differences in symptom awareness and reporting. The stratified analysis makes this concrete: the gain arose almost entirely because people with a previous diagnosis were twice as likely to have elevated burden, while ranking within each diagnosis stratum was no better than with Model A. Model A therefore remains informative as an estimate of classification performance when prior diagnostic history is unavailable.

Equally important is what machine learning did not add. Random forest and gradient boosting matched but did not exceed logistic regression, differing in AUROC by 0.003 or less, with paired confidence intervals that included zero for AUROC, AUPRC, Brier score and net benefit. The tree ensembles lost the most performance under community-grouped validation. The support vector machine, the neural network and the single decision tree, added at the request of reviewers, did not change this conclusion, and ten-fold validation reproduced the five-fold estimates. Random forest was additionally miscalibrated, with a slope of 1.25 indicating predictions compressed toward the sample mean, which is precisely the failure mode that undermines threshold-based decision rules. This reproduces, in a new population and clinical domain, the conclusion of the largest systematic comparison of the two approaches [22] and reinforces the argument that calibration deserves the same scrutiny as discrimination [23]. These findings support prioritizing the transparent nine-variable regression model for subsequent external validation, given that the more complex ensemble methods provided no measurable discrimination advantage and showed less favorable calibration or geographic transportability. We report this plainly rather than presenting the flexible models more favorably than the evidence supports. This is also why the manuscript gives most attention to logistic regression. The emphasis is a result of the comparison, not an assumption made before it: the regression model was at least as accurate as every alternative, was among the best calibrated, showed minimal optimism between training and held-out performance, and can be written as a one-line equation for use on paper or in a spreadsheet.

The methodology differs from that of most recent gastrointestinal applications of tabular machine learning. Models for *Helicobacter pylori* infection, gastric intestinal metaplasia and reflux disease published in 2025 used hospital or health-check populations and laboratory predictors [14,15,16,17]. Several evaluated performance on a single random split after synthetic oversampling, and some reported discrimination for one held-out sample without calibration or uncertainty across splits. Our design inverts several of these choices. Predictors were restricted to items obtainable in a household interview, and hyperparameters and thresholds were chosen only within training folds. Uncertainty was quantified across repeated partitions and across communities, model comparisons were paired, and clinical value was judged by net benefit. These choices produce lower but more honest estimates. They are consistent with the view that data quality and validation design, rather than algorithm choice, determine the credibility of clinical prediction models [24,45]. The study does not claim algorithmic novelty. Its contribution is a transparent, reproducible evaluation of whether such models are useful in an under-represented primary-care setting. Newer approaches such as tabular foundation models [26] could be compared against this benchmark in future work.

The modest sensitivity and F1 values reflect three features of the problem rather than a failure of model fitting. First, the predictors are eight mostly binary, self-reported items that carry limited information about a symptom outcome, so the attainable discrimination is bounded; seven very different algorithms reached a similar ceiling (AUROC 0.70 to 0.73), which indicates that the information in the predictors, not the choice of learner, limits accuracy. Second, the outcome is itself self-reported and measured once, which adds misclassification that no model can recover. Third, sensitivity and F1 depend on the chosen threshold. At the Youden threshold, sensitivity was 55.5%; at the 80% sensitivity threshold it was 80.0%, with an F1 of 0.653, at the expected cost of specificity. For these reasons we judge the models by calibration and net benefit, and we regard their role as ordering a queue rather than classifying individuals with certainty.

The dominant predictors are clinically coherent. Older age, smoking, and alcohol consumption were each independently associated with higher symptom burden, and regular physical activity, adequate hydration, and daily fiber intake were associated with lower burden, patterns consistent with the risk-factor literature for irritable bowel syndrome and related conditions [46] and with trial and cohort evidence that physical activity modifies gastrointestinal symptom severity [47,48]. Two features of our results differ from expectations derived from high-income surveys. First, sex was not associated with symptom burden, whereas most population studies report higher prevalence in women [1,46]. Second, self-reported prior diagnosis was extremely predictive, which likely reflects a mixture of genuine chronic disease and differential care-seeking. Care-seeking deserves explicit consideration. A diagnosis can be reported only by someone who consulted, so the item may partly encode access to care and willingness to seek it. Two observations argue against this being the main explanation. The frequency of previous diagnosis did not differ by education, and its association with the outcome did not vary by education or by type of health facility. Nevertheless, diagnosis was more frequent in communities served by better-resourced facilities, and our proxies for access are crude. Where diagnostic labelling depends on access, Model B could systematically under-rank people who have never been able to consult. In such settings, Model A is the more equitable choice. The temporal relationship is also uncertain. The diagnosis necessarily preceded the interview, but the questionnaire did not record when it was made or whether current symptoms began before or after it. The association is therefore best read as a marker of a recurrent or persistent symptom trajectory, not as a cause of current symptoms. It bears emphasis that this is a cross-sectional association within a classification model, not a causal claim: none of these variables can be interpreted as a modifiable cause of symptom burden from these data.

The clinical meaning of the target also deserves comment. Elevated multi-symptom burden is not a disease. It identifies people with several concurrent upper and lower gastrointestinal complaints who, in this sample, more often rated their digestive health as fair or poor and more often reported a previous diagnosis. Most of these symptoms will be functional. In meta-analysis, clinically significant endoscopic findings in people with dyspepsia were mainly erosive esophagitis (about 13%) and peptic ulcer (about 8%), and malignancy was rare [49]. For people younger than 60 years without alarm features, guidelines recommend non-invasive testing for *Helicobacter pylori* and empirical acid suppression rather than endoscopy [44]. We could not verify how many participants had an actionable condition, because no clinical examination was performed. If the proportions reported in clinical dyspepsia populations applied, roughly one in five might have an endoscopic finding of clinical relevance, mostly erosive esophagitis or peptic ulcer. This is probably an overestimate for a younger community sample. A larger proportion might benefit from *H. pylori* testing, a trial of acid suppression or lifestyle advice. The composite is therefore an appropriate target for triage to a structured primary-care assessment, but not for referral to endoscopy.

For a health system in which specialist gastroenterological assessment is effectively unavailable outside major cities, the relevant question is whether such a model could support prioritization. The decision curve analysis suggests a bounded answer. Above a threshold probability of roughly 0.25, model-guided prioritization produced greater net benefit than assessing everyone, and the advantage widened as the threshold rose, which corresponds to settings where assessment capacity is scarce relative to demand. Below that threshold, no model improved on assessing everyone, an unavoidable consequence of a condition present in 45.7% of the population. A tool of this kind is therefore best understood as a way of ordering a queue when capacity is constrained, not as a way of deciding who has a disease. The high-sensitivity operating point makes this concrete: achieving 80% sensitivity required flagging two thirds of the sample, which is a meaningful but not dramatic efficiency gain over universal assessment. In practical terms, a program that insists on detecting at least 80% of affected people would avoid only about six assessments per 100 residents, and would still miss about nine affected people per 100. Material savings, of about 14 to 23 assessments per 100, appear only when a team accepts thresholds of 0.40 to 0.50 and the loss of sensitivity that comes with them. When nearly half the population is affected, the honest conclusion is that a questionnaire-based model can usefully rank a waiting list but cannot replace clinical assessment for most symptomatic people. If capacity allows assessment of most of the population, assessing everyone may be simpler.

The subgroup analyses raise transportability and fairness considerations that are directly relevant to low-resource deployment [19,20,50,51]. A single fixed threshold behaved very differently across age bands, catching under a third of symptomatic adolescents while flagging most symptomatic adults over 40, because prevalence ranged from 31% to 57% across those strata. Discrimination was also lower in women, who made up most of the sample, than in men. If such a model were deployed with one global threshold, these differences would translate into systematically unequal detection, and adolescents would be the least well served. We do not consider these differences acceptable for a screening tool used with a single global threshold. Our nested analyses identify concrete remedies. Recalibration did not help adolescents, because their lower predicted risks were accurate. Age-specific thresholds targeting a common sensitivity of 80% equalized detection across age bands, at the cost of more false positives in younger groups. Department-level recalibration-in-the-large corrected the geographic intercept shifts. For future testing we therefore propose three steps: age-band thresholds anchored to a common sensitivity target, recalibration of the intercept to local prevalence before use in a new department, and routine monitoring of sensitivity by age and sex. Even with these adjustments, discrimination in adolescents (AUROC 0.650) remains weak, and we do not recommend the model for adolescent screening until it has been validated in that group.

The study has several strengths. The sample is comparatively large for a community survey in this region, spans five departments with contrasting geography, and contains no missing data. The validation strategy was deliberately conservative: nested cross-validation with hyperparameters and thresholds selected entirely within training folds, repeated five times, and supplemented by community-grouped validation that respects the clustered recruitment structure, as well as community-level resampling of all uncertainty estimates. Calibration, decision curves, subgroup performance and six sensitivity analyses were prespecified rather than selected after inspecting results; the additional analyses requested during peer review are identified as such, and the complete analysis pipeline is provided.

The limitations are substantial and constrain interpretation. The design is cross-sectional, so temporal ordering between exposures and symptoms cannot be established, and no causal inference is warranted. For the same reason the models classify a concurrent state and cannot be used to forecast future gastrointestinal risk. Sampling was by convenience within participating communities, so the estimates describe the sampled Honduran communities and cannot be extrapolated to the national population. The number of residents who were approached and declined participation was not recorded in the analytical database, and household identifiers were not collected, so selection bias and within-household clustering cannot be fully evaluated. The intended use population is limited to residents aged 12 to 49 years without known organic gastrointestinal disease. The results should not be extended to older adults, in whom organic disease is more frequent, or to people presenting with alarm features, who require clinical assessment regardless of any model output. All symptoms, behaviors, and prior diagnoses were self-reported and are subject to recall and social-desirability bias, which may differ between groups. Individuals who already carry a digestive diagnosis may report symptoms more readily, which could inflate the apparent value of that predictor. There is no clinical diagnostic reference standard: no endoscopy, imaging, breath testing or physician assessment was performed, so the outcome is a symptom-burden phenotype and the model is a screening and prioritization aid rather than a diagnostic device. In addition, the primary outcome was a study-defined composite symptom-burden phenotype rather than a previously validated clinical score, and the choice of a two-symptom threshold may influence both outcome prevalence and model performance. Although alternative outcome definitions and analyses of individual symptoms and symptom count yielded similar results, external validation of the phenotype remains necessary. Recruitment was geographically clustered, and although grouped validation and community-level resampling addressed this in the evaluation, residual clustering may still influence estimates. The community-level interval for the AUROC was about twice as wide as the participant-level interval, so performance in a new community may differ more than participant-level intervals suggest. Several potentially important variables were unavailable, including psychological distress, anxiety and depression, dietary detail beyond a single fiber item, medication use, body mass index, and household water and sanitation conditions, all of which are plausible contributors. All validation was internal; internal cross-validation, however carefully constructed, does not establish generalizability, and no external or temporal validation has been performed [52]. Finally, although recruitment excluded participants with objectively confirmed organic gastrointestinal disease, the predictor that carried most of the model’s signal is a participant-reported history of a digestive diagnosis that was not verified against clinical records. Its predictive value therefore depends on how consistently such diagnoses are communicated to and recalled by patients in this setting, and it may perform differently where diagnostic labelling practices differ.

Prospective evaluation is the necessary next step. Priorities are external validation in independent Honduran and regional populations, verification of the symptom phenotype against a structured Rome IV instrument administered by trained clinicians, and, if a model is deployed, a prospective assessment of whether model-guided prioritization changes the quantity or timeliness of care actually delivered, rather than only reproducing an AUROC. The staged framework for evaluating clinical prediction models, from development through external validation to impact assessment, provides the appropriate roadmap for that work [53,54].

## 5. Conclusions

In a community sample of 1838 Honduran residents aged 12 to 49 years without known organic gastrointestinal disease, elevated multi-symptom gastrointestinal burden affected 45.7% of participants and could be identified with moderate accuracy from information obtainable in a brief interview. A parsimonious, well-calibrated logistic model incorporating eight demographic and lifestyle items plus a single question about previous digestive diagnosis achieved an AUROC of 0.726 and provided net benefit over both universal and no prioritization at decision thresholds above approximately 0.25, although the efficiency gain at high sensitivity was small. More complex machine-learning approaches provided no meaningful discrimination advantage over logistic regression in this dataset, supporting the simpler and more interpretable model as the primary candidate for external validation. The resulting model should be regarded as a tool for identifying current symptom burden and ordering assessment within this age range rather than a diagnostic device or a forecast of future risk. Prospective external validation in independent populations, together with evaluation of age-specific thresholding or recalibration strategies and subgroup performance, is required before clinical implementation, and use in adolescents, older adults or people with alarm features is not supported by these data.

## Figures and Tables

**Table 1:** Participant characteristics according to elevated gastrointestinal symptom burden (*n* = 1838).

Characteristic ^1^	Overall (*n* = 1838)	No Elevated Burden (*n* = 998)	Elevated Burden (*n* = 840)	Effect Size (95% CI)	*p*
Age, years, mean (SD)	28.3 (10.9)	26.4 (10.7)	30.6 (10.6)	d = 0.39 (0.30 to 0.49)	<0.001
Age group, years, *n* (%)				Cramer V = 0.191	<0.001
12 to 17	387 (21.1)	267 (26.8)	120 (14.3)		
18 to 29	655 (35.6)	376 (37.7)	279 (33.2)		
30 to 39	443 (24.1)	202 (20.2)	241 (28.7)		
40 to 49	353 (19.2)	153 (15.3)	200 (23.8)		
Female sex, *n* (%)	1198 (65.2)	645 (64.6)	553 (65.8)	OR = 1.05 (0.87 to 1.28)	0.624
Educational attainment, *n* (%)				Cramer V = 0.108	0.002
No formal schooling	42 (2.3)	15 (1.5)	27 (3.2)		
Incomplete primary	408 (22.2)	217 (21.7)	191 (22.7)		
Complete primary	376 (20.5)	208 (20.8)	168 (20.0)		
Incomplete secondary	467 (25.4)	287 (28.8)	180 (21.4)		
Complete secondary	374 (20.3)	186 (18.6)	188 (22.4)		
Incomplete tertiary	70 (3.8)	38 (3.8)	32 (3.8)		
Complete tertiary	101 (5.5)	47 (4.7)	54 (6.4)		
Daily fiber-rich food intake, *n* (%)	1191 (64.8)	699 (70.0)	492 (58.6)	OR = 0.60 (0.50 to 0.73)	<0.001
At least eight glasses of water daily, *n* (%)	786 (42.8)	459 (46.0)	327 (38.9)	OR = 0.75 (0.62 to 0.90)	0.003
Physical activity at least three times weekly, *n* (%)	878 (47.8)	544 (54.5)	334 (39.8)	OR = 0.55 (0.46 to 0.66)	<0.001
Current cigarette smoking, *n* (%)	189 (10.3)	53 (5.3)	136 (16.2)	OR = 3.44 (2.47 to 4.80)	<0.001
Habitual alcohol consumption, *n* (%)	300 (16.3)	100 (10.0)	200 (23.8)	OR = 2.81 (2.16 to 3.64)	<0.001
Previously diagnosed digestive disorder, *n* (%)	399 (21.7)	89 (8.9)	310 (36.9)	OR = 5.97 (4.61 to 7.74)	<0.001
Department, *n* (%)				Cramer V = 0.112	<0.001
Colón	550 (29.9)	283 (28.4)	267 (31.8)		
Comayagua	490 (26.7)	243 (24.3)	247 (29.4)		
El Paraíso	53 (2.9)	26 (2.6)	27 (3.2)		
Intibucá	585 (31.8)	365 (36.6)	220 (26.2)		
Olancho	160 (8.7)	81 (8.1)	79 (9.4)		
Self-rated digestive health, *n* (%)				Cramer V = 0.503	<0.001
Excellent	427 (23.2)	371 (37.2)	56 (6.7)		
Good	688 (37.4)	438 (43.9)	250 (29.8)		
Fair	588 (32.0)	181 (18.1)	407 (48.5)		
Poor	102 (5.5)	7 (0.7)	95 (11.3)		
Very poor	33 (1.8)	1 (0.1)	32 (3.8)		

^1^ Elevated gastrointestinal symptom burden is defined as at least two of four current symptom indicators. Percentages are column percentages within each outcome group. Effect sizes are Cohen’s d for continuous variables, odds ratios for binary variables and Cramer’s V for multi-category variables. *p* values are from the Welch *t* test or the chi-square test (with continuity correction for 2 × 2 tables). Self-rated digestive health is shown for descriptive purposes and was not used as a predictor. CI, confidence interval; OR, odds ratio; SD, standard deviation.

**Table 2:** Prevalence of gastrointestinal symptoms, symptom burden and self-reported digestive diagnoses (*n* = 1838).

Indicator ^1^	*n*	%	95% CI
Individual symptom indicators			
Abdominal pain, previous 30 days	596	32.4	30.3 to 34.6
Heartburn, previous 2 weeks	570	31.0	28.9 to 33.2
Any gastroesophageal reflux above “never”	1017	55.3	53.1 to 57.6
Frequent or constant reflux	195	10.6	9.3 to 12.1
Post-prandial abdominal bloating	587	31.9	29.8 to 34.1
Composite symptom burden			
Elevated burden, at least 2 of 4 indicators (primary outcome)	840	45.7	43.4 to 48.0
High burden, at least 3 of 4 indicators	453	24.6	22.7 to 26.7
Reflux frequency			
Never	821	44.7	42.4 to 46.9
Rarely	457	24.9	22.9 to 26.9
Sometimes	365	19.9	18.1 to 21.7
Frequently	149	8.1	6.9 to 9.4
Always	46	2.5	1.9 to 3.3
Self-reported previous digestive diagnosis			
Any previous digestive diagnosis (harmonized)	399	21.7	19.9 to 23.7
*Helicobacter pylori* infection	139	7.6	6.4 to 8.9
Gastritis	136	7.4	6.3 to 8.7
Gastroesophageal reflux disease	69	3.8	3.0 to 4.7
Irritable bowel syndrome	47	2.6	1.9 to 3.4
Colitis	23	1.3	0.8 to 1.9
Other digestive condition	20	1.1	0.7 to 1.7

^1^ Confidence intervals are Wilson intervals for a single proportion. Specific diagnoses are not mutually exclusive. The harmonized diagnosis variable is an affirmative response to the global diagnosis item or to at least one specific diagnosis. None of these categories represents a clinically verified diagnosis; all are participant-reported.

**Table 3:** Comparative performance of candidate classification models under repeated nested cross-validation (n = 1838, 840 events).

Model ^1^	AUROC (95% CI)	AUPRC (95% CI)	Sensitivity (95% CI)	Specificity (95% CI)	PPV (95% CI)	NPV (95% CI)	F1	Balanced Accuracy	Brier (95% CI)	Calibration Slope (95% CI)
Model A: demographic and lifestyle										
Logistic regression	0.663 (0.639 to 0.688)	0.626 (0.593 to 0.662)	0.517 (0.483 to 0.552)	0.706 (0.680 to 0.735)	0.597 (0.564 to 0.633)	0.635 (0.607 to 0.663)	0.554	0.612	0.227 (0.221 to 0.234)	0.939 (0.791 to 1.104)
Elastic-net logistic regression	0.663 (0.639 to 0.688)	0.626 (0.593 to 0.661)	0.513 (0.479 to 0.549)	0.711 (0.684 to 0.739)	0.599 (0.566 to 0.635)	0.634 (0.607 to 0.663)	0.553	0.612	0.227 (0.221 to 0.234)	0.957 (0.805 to 1.126)
Random forest	0.660 (0.636 to 0.685)	0.626 (0.592 to 0.663)	0.494 (0.461 to 0.530)	0.734 (0.706 to 0.762)	0.610 (0.575 to 0.647)	0.633 (0.605 to 0.659)	0.546	0.614	0.228 (0.223 to 0.233)	1.290 (1.090 to 1.519)
Gradient boosting (XGBoost)	0.661 (0.636 to 0.686)	0.633 (0.598 to 0.668)	0.463 (0.430 to 0.499)	0.757 (0.730 to 0.783)	0.616 (0.578 to 0.654)	0.626 (0.600 to 0.654)	0.529	0.610	0.227 (0.220 to 0.233)	0.869 (0.735 to 1.016)
Model B: clinically augmented										
Logistic regression	0.726 (0.702 to 0.748)	0.705 (0.674 to 0.737)	0.555 (0.521 to 0.588)	0.798 (0.773 to 0.822)	0.698 (0.664 to 0.732)	0.680 (0.653 to 0.707)	0.618	0.676	0.208 (0.200 to 0.216)	0.955 (0.838 to 1.081)
Elastic-net logistic regression	0.725 (0.702 to 0.748)	0.705 (0.674 to 0.737)	0.556 (0.523 to 0.590)	0.799 (0.774 to 0.823)	0.699 (0.666 to 0.734)	0.681 (0.654 to 0.707)	0.619	0.677	0.208 (0.200 to 0.216)	0.981 (0.861 to 1.113)
Random forest	0.723 (0.699 to 0.746)	0.692 (0.659 to 0.727)	0.545 (0.512 to 0.580)	0.805 (0.781 to 0.830)	0.701 (0.667 to 0.735)	0.678 (0.650 to 0.704)	0.614	0.675	0.209 (0.203 to 0.216)	1.254 (1.104 to 1.420)
Gradient boosting (XGBoost)	0.725 (0.700 to 0.748)	0.708 (0.678 to 0.741)	0.537 (0.502 to 0.572)	0.827 (0.804 to 0.850)	0.723 (0.688 to 0.757)	0.680 (0.653 to 0.706)	0.616	0.682	0.206 (0.198 to 0.215)	0.930 (0.825 to 1.047)
Model B: additional classifiers										
Decision tree	0.704 (0.679 to 0.728)	0.674 (0.640 to 0.710)	0.488 (0.454 to 0.522)	0.854 (0.833 to 0.876)	0.737 (0.704 to 0.774)	0.665 (0.639 to 0.690)	0.587	0.671	0.212 (0.204 to 0.220)	0.927 (0.816 to 1.055)
Support vector machine	0.720 (0.697 to 0.743)	0.701 (0.670 to 0.732)	0.525 (0.492 to 0.558)	0.831 (0.808 to 0.854)	0.723 (0.690 to 0.758)	0.675 (0.649 to 0.701)	0.608	0.678	0.210 (0.202 to 0.218)	0.917 (0.808 to 1.036)
Neural network (multilayer perceptron)	0.713 (0.689 to 0.737)	0.682 (0.650 to 0.715)	0.561 (0.527 to 0.595)	0.792 (0.767 to 0.816)	0.694 (0.660 to 0.728)	0.682 (0.655 to 0.707)	0.620	0.676	0.212 (0.204 to 0.219)	1.052 (0.919 to 1.190)
Community-grouped cross-validation										
Model A, logistic regression	0.656 (0.631 to 0.681)	0.623							0.229	0.887
Model B, logistic regression	0.722 (0.698 to 0.745)	0.704							0.208	0.948
Model B, random forest	0.711 (0.686 to 0.735)	0.695							0.212	1.178
Model B, gradient boosting (XGBoost)	0.712 (0.688 to 0.735)	0.706							0.211	0.839

^1^ All estimates are derived from out-of-fold predictions averaged across five repetitions of stratified five-fold cross-validation, with hyperparameters and classification thresholds selected exclusively within training folds. Confidence intervals are percentile bootstrap intervals based on 2000 resamples. Threshold-dependent metrics use the Youden threshold fixed in the training folds (0.473 to 0.503 depending on model). Calibration intercepts were 0.000 to 0.007 for all principal models. Community-grouped cross-validation used StratifiedGroupKFold over 85 communities with three repetitions; full metrics are given in Supplementary Table S3. The decision tree, support vector machine and neural network were added in revision; their Model A results are given in Supplementary Table S11. Community-level cluster bootstrap intervals are given in Table 4. All validation is internal. AUPRC, area under the precision–recall curve; AUROC, area under the receiver operating characteristic curve; NPV, negative predictive value; PPV, positive predictive value.

**Table 4:** Paired differences in performance between Model B logistic regression and comparator models, with participant-level and community-level (cluster) bootstrap 95% confidence intervals.

Comparison ^1^	AUROC Difference	DeLong *p*	AUPRC Difference	Brier Difference	NB30 Difference	NB40 Difference	NB50 Difference
vs. elastic-net logistic (Model B)	0.0005 (0.0000 to 0.0010) [−0.0000 to 0.0012]	0.036	0.000 (−0.001 to 0.001) [−0.001 to 0.002]	−0.0001 (−0.0003 to 0.0001) [−0.0004 to 0.0003]	0.001 (−0.003 to 0.004) [−0.003 to 0.005]	0.004 (0.002 to 0.007) [0.002 to 0.007]	−0.001 (−0.003 to 0.001) [−0.004 to 0.001]
vs. random forest (Model B)	0.0032 (−0.0043 to 0.0108) [−0.0064 to 0.0143]	0.410	0.013 (0.003 to 0.022) [0.001 to 0.024]	−0.0020 (−0.0043 to 0.0004) [−0.0049 to 0.0010]	−0.001 (−0.011 to 0.009) [−0.011 to 0.008]	0.004 (−0.009 to 0.017) [−0.011 to 0.020]	0.001 (−0.009 to 0.009) [−0.007 to 0.009]
vs. gradient boosting (Model B)	0.0007 (−0.0076 to 0.0097) [−0.0110 to 0.0116]	0.871	−0.003 (−0.014 to 0.007) [−0.016 to 0.008]	0.0010 (−0.0016 to 0.0036) [−0.0022 to 0.0045]	−0.004 (−0.014 to 0.007) [−0.015 to 0.007]	0.007 (−0.005 to 0.019) [−0.005 to 0.019]	−0.004 (−0.015 to 0.006) [−0.014 to 0.007]
vs. decision tree (Model B)	0.0219 (0.0111 to 0.0326) [0.0105 to 0.0358]	<0.001	0.031 (0.018 to 0.042) [0.017 to 0.044]	−0.0045 (−0.0073 to −0.0017) [−0.0079 to −0.0013]	0.005 (−0.006 to 0.015) [−0.003 to 0.012]	0.023 (0.010 to 0.035) [0.008 to 0.036]	0.004 (−0.007 to 0.014) [−0.005 to 0.014]
vs. support vector machine (Model B)	0.0053 (−0.0024 to 0.0129) [−0.0023 to 0.0128]	0.155	0.005 (−0.001 to 0.011) [−0.001 to 0.010]	−0.0023 (−0.0048 to 0.0001) [−0.0054 to 0.0011]	−0.007 (−0.015 to 0.002) [−0.018 to 0.005]	0.010 (−0.004 to 0.024) [−0.011 to 0.028]	−0.003 (−0.011 to 0.005) [−0.012 to 0.005]
vs. neural network (Model B)	0.0124 (0.0039 to 0.0210) [0.0039 to 0.0198]	0.003	0.023 (0.009 to 0.038) [0.006 to 0.037]	−0.0043 (−0.0073 to −0.0015) [−0.0069 to −0.0008]	−0.004 (−0.012 to 0.005) [−0.011 to 0.003]	0.013 (0.001 to 0.026) [0.002 to 0.025]	0.007 (−0.005 to 0.018) [−0.001 to 0.016]
vs. logistic regression, Model A	0.0627 (0.0456 to 0.0793) [0.0437 to 0.0841]	<0.001	0.079 (0.056 to 0.102) [0.046 to 0.112]	−0.0199 (−0.0259 to −0.0138) [−0.0267 to −0.0131]	0.006 (−0.005 to 0.018) [−0.004 to 0.016]	0.040 (0.022 to 0.057) [0.018 to 0.066]	0.057 (0.035 to 0.079) [0.032 to 0.084]

^1^ Differences are logistic regression (Model B) minus comparator; positive values favor logistic regression for AUROC, AUPRC and net benefit, and negative values favor it for the Brier score. Each difference was computed on the same 2000 bootstrap resamples of the averaged out-of-fold predictions. Resampling either of participants (first interval) or of the 85 communities (second interval, in brackets) was carried out. *p* values for the AUROC are from the DeLong test. NB30, NB40 and NB50 denote net benefit at threshold probabilities of 0.30, 0.40 and 0.50. AUPRC, area under the precision–recall curve; AUROC, area under the receiver operating characteristic curve.

**Table 5:** Operating characteristics of Model B logistic regression under alternative threshold strategies, overall and by age band.

Strategy ^1^	Threshold	Flagged, %	Sensitivity, %	Specificity, %	PPV, %	Sensitivity by Age Band, %	Net Reduction per 100
Youden index, global (nested)	0.475	36.3	55.5	79.8	69.8	32/46/64/74	20.9
80% sensitivity, global (nested)	0.326	66.3	80.0	45.3	55.2	49/74/90/95	5.7
Youden index, age-specific (nested)	0.43/0.42/0.52/0.62	33.1	51.4	82.4	71.1	32/51/57/57	n/a
80% sensitivity, age-specific (nested)	0.24/0.31/0.38/0.43	68.6	80.1	41.1	53.4	82/80/79/80	n/a
Age-band recalibration plus global Youden (nested)	0.475	36.1	55.1	80.0	69.8	32/46/66/70	n/a
Capacity: highest 30% of predicted risk	0.535	30.0	48.9	85.9	74.5	30/40/56/64	26.4
Capacity: highest 50% of predicted risk	0.386	50.0	67.0	64.3	61.3	38/56/79/86	11.0
Harm ratio 2.3:1 (threshold probability 0.30)	0.300	74.3	85.4	35.1	52.5	53/81/95/98	3.4
Harm ratio 1.5:1 (threshold probability 0.40)	0.400	47.2	65.7	68.3	63.6	37/54/78/84	13.6
Harm ratio 1.0:1 (threshold probability 0.50)	0.500	33.1	52.1	83.0	72.0	32/44/59/68	23.2

^1^ Strategies labelled “nested” use thresholds derived in the training folds and applied unchanged to held-out folds; capacity and harm-ratio thresholds are located on the out-of-fold predictions and are illustrative. Net reduction is the number of assessments avoided per 100 people relative to assessing everyone, at equal net benefit [38]. Sensitivity by age band is shown for 12 to 17, 18 to 29, 30 to 39 and 40 to 49 years. The harm ratio is the number of unnecessary assessments considered acceptable to detect one additional person with elevated burden. n/a, not applicable.

**Table 6:** Unadjusted and adjusted odds ratios for elevated gastrointestinal symptom burden from the Model B logistic regression (*n* = 1838).

Variable ^1^	Unadjusted OR (95% CI)	*p*	Adjusted OR (95% CI)	*p*	Adjusted OR, Cluster-Robust 95% CI
Age, per 10 years	1.44 (1.32 to 1.57)	<0.001	1.20 (1.09 to 1.33)	<0.001	1.00 to 1.44
Female sex	1.05 (0.87 to 1.28)	0.589	1.21 (0.97 to 1.51)	0.099	0.97 to 1.50
Educational attainment, per category	1.01 (0.95 to 1.08)	0.697	1.01 (0.94 to 1.09)	0.706	0.94 to 1.09
Daily fiber-rich food intake	0.60 (0.50 to 0.73)	<0.001	0.78 (0.63 to 0.97)	0.025	0.57 to 1.07
At least eight glasses of water daily	0.75 (0.62 to 0.90)	0.002	0.74 (0.60 to 0.92)	0.005	0.62 to 0.89
Physical activity at least three times weekly	0.55 (0.46 to 0.66)	<0.001	0.68 (0.55 to 0.84)	<0.001	0.50 to 0.92
Current cigarette smoking	3.44 (2.47 to 4.80)	<0.001	1.99 (1.35 to 2.92)	<0.001	1.30 to 3.04
Habitual alcohol consumption	2.81 (2.16 to 3.64)	<0.001	2.08 (1.52 to 2.84)	<0.001	1.53 to 2.82
Previously diagnosed digestive disorder	5.97 (4.61 to 7.74)	<0.001	5.13 (3.93 to 6.71)	<0.001	3.61 to 7.30

^1^ Adjusted estimates are mutually adjusted for all listed variables. Educational attainment is modelled as an ordinal score from 0 (no formal schooling) to 6 (completed tertiary education). Model likelihood ratio *p* < 0.001; McFadden pseudo R-squared 0.134. Cluster-robust intervals use a sandwich variance estimator with the 85 communities as clusters. These are cross-sectional associations and do not support causal interpretation. CI, confidence interval; OR, odds ratio.

**Table 7:** Subgroup and geographic performance of the selected model (Model B, logistic regression).

Subgroup ^1^	*n*	Events	Prevalence	AUROC (95% CI)	AUPRC	Sensitivity	Specificity	PPV	Brier	Calibration Intercept	Calibration Slope
Overall	1838	840	0.457	0.726 (0.701 to 0.748)	0.705	0.555	0.798	0.698	0.208	−0.000	0.955
Female	1198	553	0.462	0.703 (0.673 to 0.731)	0.699	0.515	0.802	0.690	0.214	−0.002	0.942
Male	640	287	0.448	0.759 (0.721 to 0.796)	0.719	0.631	0.790	0.710	0.196	0.003	0.975
Age 12 to 17 years	387	120	0.310	0.650 (0.589 to 0.718)	0.512	0.317	0.933	0.679	0.192	−0.076	1.009
Age 18 to 29 years	655	279	0.426	0.707 (0.664 to 0.750)	0.659	0.455	0.867	0.718	0.210	0.050	1.041
Age 30 to 39 years	443	241	0.544	0.706 (0.658 to 0.755)	0.743	0.639	0.673	0.700	0.216	0.047	0.841
Age 40 to 49 years	353	200	0.567	0.719 (0.666 to 0.768)	0.783	0.735	0.556	0.684	0.209	−0.079	0.907
Colón	550	267	0.485	0.665 (0.617 to 0.709)	0.685	0.506	0.746	0.652	0.226	0.080	0.761
Comayagua	490	247	0.504	0.794 (0.753 to 0.835)	0.775	0.692	0.765	0.750	0.189	−0.006	1.071
El Paraíso	53	27	0.509	0.848 (0.730 to 0.939)	0.861	0.519	0.923	0.875	0.184	0.467	2.073
Intibucá	585	220	0.376	0.722 (0.678 to 0.765)	0.640	0.500	0.841	0.655	0.201	−0.242	1.054
Olancho	160	79	0.494	0.665 (0.584 to 0.751)	0.687	0.456	0.840	0.735	0.235	0.457	0.773

^1^ Metrics are computed within subgroups from the same out-of-fold predictions, using the single overall Youden threshold of 0.475. Confidence intervals for the AUROC are percentile bootstrap intervals based on 1000 resamples. The calibration intercept (calibration-in-the-large) indicates whether average predicted risk matches observed prevalence within the subgroup: 0 is ideal, negative values indicate overestimation and positive values underestimation. The calibration slope indicates whether predictions are too extreme or too moderate within the subgroup: 1 is ideal, values below 1 indicate predictions that are too extreme and values above 1 predictions that are too compressed. Within small subgroups both quantities are imprecise. Estimates for El Paraíso are based on 53 participants and are unstable; the calibration slope of 2.07 in that department should not be interpreted as evidence of superior calibration. AUPRC, area under the precision–recall curve; AUROC, area under the receiver operating characteristic curve; PPV, positive predictive value.

**Table 8:** Discrimination of Model A and Model B by previous digestive diagnosis and by proxies for access to care (logistic regression, out-of-fold predictions).

Subgroup ^1^	*n*	Prevalence	Previous Diagnosis, %	AUROC Model A (95% CI)	AUROC Model B (95% CI)	Difference B Minus A (95% CI)
All participants	1838	0.457	21.7	0.663 (0.639 to 0.689)	0.726 (0.701 to 0.748)	0.063 (0.046 to 0.080)
No previous digestive diagnosis	1439	0.368	0.0	0.647 (0.616 to 0.678)	0.643 (0.611 to 0.674)	−0.004 (−0.008 to 0.000)
Previous digestive diagnosis	399	0.777	100.0	0.626 (0.566 to 0.692)	0.621 (0.560 to 0.689)	−0.005 (−0.014 to 0.003)
Education: primary or less	826	0.467	21.5	0.660 (0.619 to 0.696)	0.719 (0.682 to 0.752)	0.059 (0.034 to 0.084)
Education: secondary or higher	1012	0.449	21.8	0.665 (0.631 to 0.700)	0.731 (0.696 to 0.760)	0.066 (0.044 to 0.088)
Community served by a CIS	1218	0.497	25.5	0.710 (0.680 to 0.738)	0.762 (0.734 to 0.786)	0.052 (0.033 to 0.070)
Community served by another unit	620	0.379	14.4	0.548 (0.500 to 0.593)	0.625 (0.577 to 0.669)	0.077 (0.044 to 0.112)

^1^ Percentile bootstrap 95% confidence intervals (1000 resamples); the AUROC difference is paired within each subgroup. Within the previous-diagnosis strata the predictor is constant, so the comparison shows how well the remaining eight items rank participants within each stratum. Facility type refers to the primary-care facility serving the participant’s community and is used only as an approximate marker of access to care. AUROC, area under the receiver operating characteristic curve; CIS, Centro Integral de Salud.

## Data Availability

The complete analysis code that reproduces every reported result is provided as Supplementary Material. The de-identified analytical dataset is available from the corresponding author upon reasonable request, subject to authorization by the Comité de Ética en Investigación Científica en Salud of the Colegio de Nutricionistas y Dietistas de Honduras. Individual-level records are not publicly deposited because participants did not consent to open publication and because ethical approval requires the data to be held on secure institutional servers.

## Supplementary Materials

The following supporting information can be downloaded at https://www.mdpi.com/article/10.3390/diagnostics16213440/s1. Table S1, Variable definitions, coding rules and complete recoding log; Table S2, Hyperparameter search spaces and configurations selected during nested cross-validation; Table S3, Cross-validation performance by repetition and by validation strategy; Table S4, Sensitivity analyses using alternative outcome definitions and alternative analytical samples; Table S5, Extended subgroup performance; Table S6, TRIPOD+AI checklist mapped to manuscript sections; Table S7, STROBE cross-sectional checklist mapped to manuscript sections; Table S8, Models for individual symptom indicators and ordinal model for symptom count; Table S9, Full Model B logistic regression equation; Table S10, Fold-level performance, ten-fold cross-validation and optimism; Table S11, Additional classifiers (decision tree, support vector machine, neural network); Table S12, PROBAST+AI self-assessment; Table S13, Participant-level and community-level bootstrap confidence intervals; Figure S1, Calibration plots for all candidate models; Figure S2, SHAP dependence plots for the four highest-ranked variables; Figure S3, Distribution of fold-level AUROC under five-fold and ten-fold cross-validation; File S1, Analysis code and AI-model development and validation protocol.

## Abbreviations

The following abbreviations are used in this manuscript:

AI	Artificial intelligence
AUPRC	Area under the precision–recall curve
AUROC	Area under the receiver operating characteristic curve
CEICS	Comité de Ética en Investigación Científica en Salud, Colegio de Nutricionistas y Dietistas de Honduras
CI	Confidence interval
CIS	Centro Integral de Salud
CIOMS	Council for International Organizations of Medical Sciences
ICC	Intraclass correlation coefficient
LR	Logistic regression
MLP	Multilayer perceptron (feed-forward neural network)
NPV	Negative predictive value
OR	Odds ratio
PAHO	Pan American Health Organization
PPV	Positive predictive value
PROBAST + AI	Prediction model Risk Of Bias Assessment Tool, artificial intelligence extension
ROC	Receiver operating characteristic
SD	Standard deviation
SHAP	SHapley Additive exPlanations
STROBE	Strengthening the Reporting of Observational Studies in Epidemiology
SVM	Support vector machine
TRIPOD+AI	Transparent Reporting of a multivariable prediction model for Individual Prognosis Or Diagnosis, artificial intelligence extension
WHO	World Health Organization
WMA	World Medical Association
XGBoost	Extreme Gradient Boosting
