# Supplementary material for: Explainable Classification Models for Identifying Elevated Gastrointestinal Symptom Burden in Honduran Communities: A Cross-Sectional Study
Source: Diagnostics (Basel). 2026 Oct 22;16(21):3440. doi: 10.3390/diagnostics16213440 (PMC13761740; doi:10.3390/diagnostics16213440)
Supplement: Supplementary file 1 [file diagnostics-16-03440-s001.zip › File_S1_AI_Development_and_Validation_Protocol.pdf]

File S1. AI Model Development and Validation Protocol

Study: Explainable Classification Models for Identifying Elevated Gastrointestinal Symptom Burden in Honduran Communities: A Cross-Sectional Study. This protocol documents every analytical decision used to develop, validate and explain the models, so that the pipeline can be audited and reproduced. It complements the TRIPOD+AI (Table S6), STROBE (Table S7) and PROBAST+AI (Table S12) checklists.

1. Intended use and model type

The models estimate the probability that elevated multi-symptom gastrointestinal burden is present at the time of a community interview. They are concurrent (diagnostic-type) classification models in TRIPOD terminology, intended to order a queue for structured primary-care assessment. They are not diagnostic devices, they do not forecast future risk, and they are not intended for people with alarm features, people older than 49 years, or people with known organic gastrointestinal disease.

2. Data source and governance

Source: one participant-level spreadsheet (1838 records, 33 variables) from a cross-sectional community survey in five Honduran departments (18 September to 19 November 2025). Ethics: Comité de Ética en Investigación Científica en Salud, Colegio de Nutricionistas y Dietistas de Honduras (CEICS-cndh 002-10-2025). Administrative scheduling fields, the data-collector name and the facility name were removed before analysis. Data are held on institutional servers; no record leaves the analysis environment.

3. Data audit and preprocessing

(a) Structural audit: counts of records, variables, missing cells and out-of-range values. (b) Whitespace normalization of text fields. (c) Harmonization of inconsistent health-region codes from department, with the original field retained. (d) Flagging, without deletion, of out-of-window dates and of records identical across all 31 substantive fields. (e) Harmonization of the previous-diagnosis items (affirmative on either the global item or the checklist). (f) No imputation, because there were no missing values. Every rule is logged in Table S1.

4. Outcome and predictors

Primary outcome: at least two of four current symptom indicators (abdominal pain in 30 days, heartburn in two weeks, any reflux above "never", post-prandial bloating). Secondary endpoints: each indicator separately, strict reflux, and the 0 to 4 symptom count. Sensitivity outcomes: at least three indicators, strict reflux definition, self-rated digestive health below "good". Model A predictors: age, sex, education (ordinal 0 to 6), daily fibre, at least eight glasses of water, physical activity at least three times weekly, current smoking, habitual alcohol. Model B: Model A plus previous digestive diagnosis. Outcome components and self-rated digestive health are never used as predictors (leakage control). Geography and facility type are used only for grouping, subgroup and cluster analyses.

5. Algorithms and hyperparameter spaces

Logistic regression (unpenalized, standardized inputs); elastic-net logistic regression (C in {0.01, 0.1, 1}, l1 ratio 0.5); random forest (300 trees; depth {4, none}; minimum leaf {10, 50}; features per split {sqrt, 0.5}); XGBoost (300 trees, learning rate 0.05, subsample 0.8, column sample 0.8; depth {2, 3}; minimum child weight {5, 20}; L2 penalty {1, 5}); decision tree (depth {2 to 5}; minimum leaf {20, 50}); support vector machine (RBF kernel, Platt scaling; C {0.1, 1, 10}; gamma {scale, 0.01}); multilayer perceptron (hidden layers {(8), (16, 8)}; L2 penalty {0.001, 0.1}; early stopping). No oversampling or class weighting (prevalence 45.7%).

6. Validation design

Primary: nested cross-validation with five outer stratified folds (80% development, 20% evaluation) repeated five times with seeds 20251212 to 20251216, and an inner three-fold stratified grid search maximizing AUROC. Classification thresholds (Youden index and 80% sensitivity) are derived from inner cross-validated predictions in each outer training fold and applied unchanged to the held-out fold. Secondary: the same nested design with ten outer folds (90% and 10%), and community-grouped nested cross-validation (StratifiedGroupKFold, three repetitions) so that no community contributes to both development and evaluation. No information from evaluation folds is used for tuning, threshold selection or recalibration. All validation is internal.

7. Performance evaluation

Metrics: AUROC, AUPRC, sensitivity, specificity, PPV, NPV, F1, balanced accuracy, Brier score, calibration intercept (offset model) and slope. Predictions are averaged across repetitions; summary thresholds are the mean of fold-specific thresholds. Uncertainty: percentile bootstrap (2000 resamples) at participant level and at community level (resampling the 85 communities); fold-level mean, standard deviation and t-based interval; apparent minus held-out AUROC as optimism. Model comparison: paired bootstrap differences in AUROC, AUPRC, Brier score and net benefit at thresholds 0.30, 0.40 and 0.50, plus the DeLong test.

8. Clinical utility and operating points

Decision curve analysis over threshold probabilities 0.05 to 0.70 against assess-all and assess-none strategies, reported also as net assessments avoided per 100 people. Operating points: Youden (reference), nested 80% sensitivity, capacity-anchored (20% to 70% assessed) and harm-ratio thresholds (0.25 to 0.50).

9. Explainability

Logistic regression: adjusted odds ratios with model-based and community cluster-robust intervals, forest plot, exact linear SHAP values and the full equation. Gradient boosting: TreeSHAP on the model refitted to all data with the modal nested configuration; global importance as mean absolute SHAP; direction as Spearman correlation between feature and SHAP value. SHAP values describe model behaviour and are never interpreted causally.

10. Fairness and subgroup evaluation

Subgroups: sex, age band (12 to 17, 18 to 29, 30 to 39, 40 to 49 years), department, previous diagnosis, education and facility type (access proxies). Minimum of 10 events and 10 non-events per subgroup. Reported: AUROC with bootstrap interval, AUPRC, sensitivity, specificity, PPV, Brier score, calibration intercept and slope, and Model B minus Model A AUROC. Mitigation strategies tested within the nested procedure: age-specific Youden and 80%-sensitivity thresholds, and recalibration-in-the-large by age band and by department.

11. Sensitivity analyses

Alternative outcomes (at least three indicators; strict reflux; self-rated health), exclusion of redundant identical records, exclusion of out-of-window dates, and the unharmonized previous-diagnosis item.

12. Reproducibility

Single seed (20251212) for all stochastic components. Software: Python 3.13, pandas 3.0.5, NumPy 2.5.3, SciPy 1.18.1, statsmodels 0.15.0, scikit-learn 1.9.1, XGBoost 3.4.1, SHAP 0.53.0, Matplotlib 3.11.2. Hardware: CPU-only Linux workstation (Intel Xeon, 2.1 GHz, two cores, 8 GB RAM); no GPU. The accompanying script (analysis_pipeline.py) regenerates every table, figure and number from the source spreadsheet.

13. Conditions before any clinical use

(a) External validation in independent Honduran and regional populations, with a sample size calculated for validation of calibration and net benefit. (b) Verification of the outcome against a clinician-administered Rome

IV assessment. (c) Local recalibration of the intercept to the prevalence of each new setting. (d) Age-band thresholds anchored to an agreed sensitivity target. (e) Prospective impact evaluation of whether prioritization changes timeliness or quantity of care. (f) Routine monitoring of sensitivity and calibration by age, sex and locality, with predefined triggers for recalibration.
