# Supplementary material for: Explainable Classification Models for Identifying Elevated Gastrointestinal Symptom Burden in Honduran Communities: A Cross-Sectional Study
Source: Diagnostics (Basel). 2026 Oct 22;16(21):3440. doi: 10.3390/diagnostics16213440 (PMC13761740; doi:10.3390/diagnostics16213440)
Supplement: Supplementary file 1 [file diagnostics-16-03440-s001.zip › Supplementary_Materials.pdf]

Supplementary Materials

Explainable Classification Models for Identifying Elevated Gastrointestinal Symptom Burden in Honduran Communities: A Cross-Sectional Study

Table S1. Variable definitions, coding rules and complete recoding log

S1a. Analytical variables

Analytical name	Source field	Definition and coding	Role
age	Edadenanos	Age in completed years, integer, range 12 to 49	Predictor (Models A and B)
age_cat	derived	12 to 17, 18 to 29, 30 to 39, 40 to 49 years	Subgroup analysis only
female	Sexo	1 = Mujer, 0 = Hombre	Predictor (A and B)
edu_ord	Niveleducativo	Ordinal 0 to 6: no formal schooling, incomplete primary, complete primary, incomplete secondary, complete secondary, incomplete tertiary, complete tertiary	Predictor (A and B)
fiber_daily	Consumealimentricosenfibra	1 = daily consumption of fibre-rich foods	Predictor (A and B)
water_8	Bebealmenos8vasosdeagua	1 = at least eight glasses of water daily	Predictor (A and B)
pa_3wk	Realizaactividadfisica3vecesxs	1 = physical activity at least three times weekly	Predictor (A and B)
smoke	Fumacigarrillosactualmente	1 = current cigarette smoking	Predictor (A and B)
alcohol	Consumealcoholhabitualmente	1 = habitual alcohol consumption	Predictor (A and B)
prior_dx	Harecibidodiagosticomedico + six specific-diagnosis fields	1 if the global item is affirmative or any specific diagnosis is affirmative	Predictor (Model B only)
sx_pain	Hapresentadodolorabd30dias	1 = abdominal pain in previous 30 days	Outcome component; secondary endpoint
sx_heartburn	acidezestomacalultimas2S	1 = heartburn in previous 2 weeks	Outcome component; secondary endpoint
sx_reflux_any	Frecuenciareflujogastrico	1 if response is above "Nunca"	Outcome component; secondary endpoint
sx_reflux_strict	Frecuenciareflujogastrico	1 if response is "Frecuentemente" or "Siempre"	Sensitivity outcome component; secondary endpoint
sx_bloating	Inflamacionabdominaltrascom	1 = post-prandial abdominal bloating	Outcome component; secondary endpoint
sx_count	derived	Sum of the four current symptom indicators, 0 to 4	Intermediate; ordinal secondary endpoint
outcome	derived	1 if sx_count >= 2	**Primary outcome**
outcome_ge3	derived	1 if sx_count >= 3	Sensitivity analysis 1
outcome_strict	derived	1 if strict-reflux symptom count >= 2	Sensitivity analysis 2
outcome_selfrated	Sintomasdigestcomocalificaria	1 if Regular, Malo or Muy malo	Sensitivity analysis 3
department, municipality, community	Departamento, Municipio, Nombredecomunidad	Whitespace-normalized text	Subgroup and grouped validation only; cluster bootstrap unit (community)
facility_type	Nombredelestablecimientodesalud (type prefix only)	Centro Integral de Salud versus other primary-care unit	Access-to-care proxy in subgroup analysis only; never a predictor
RegionSanitaria_corr	derived	Health region harmonized to department	Documentation only
dup_excess	derived	1 for redundant copies within groups of identical records	Sensitivity analysis 4
date_out_of_window	Fecharecolecciondelainfo	1 if date outside 18 Sep to 19 Nov 2025	Sensitivity analysis 5

S1b. Variables excluded from the analytical dataset

Source field	Reason for exclusion
Three administrative fieldwork-scheduling identifiers	Administrative variables describing how fieldwork was organized; no clinical meaning
Free-text data-collector name field	Identifies the individual data collector; removed for privacy and to prevent collector-level leakage
Nombredelestablecimientodesalud	Health facility name; encodes collection logistics rather than a transportable clinical characteristic (only its type prefix was used, as an access proxy, in subgroup analyses)
No Registro de cada BD	Secondary record counter, redundant
ReginSanitaria (raw)	Superseded by the harmonized region variable

Source field	Reason for exclusion
Especifiqueotro	Free-text field, 1805 of 1838 values were "NINGUNO"; retained descriptively only
Sintomasdigestcomocalificaria	Global subjective rating of the same symptom experience as the outcome; excluded from predictors to prevent outcome leakage

S1c. Recoding log

1. **Whitespace normalization.** Leading, trailing and repeated internal whitespace removed from all text fields. This collapsed two label variants: one municipality (99 + 51 records) and one health facility (88 + 2 records).

2. **Health-region harmonization.** Seven records (source IDs 614, 631, 845, 873, 955, 1123, 1603) carried region code RS1 (Atlántida), although department, municipality, community and health facility were mutually consistent with Colón (n = 2), Comayagua (n = 1) or Intibucá (n = 4). No sampled community lies in Atlántida. A harmonized region variable was created from department; the original field was retained unmodified.

3. **Collection dates.** Two records (source IDs 322, dated 18 April 2025, and 954, dated 12 July 2025) fell outside the principal fieldwork window. Dates were not overwritten. The date variable was excluded from modelling and a sensitivity analysis excluding these records is reported.

4. **Indistinguishable records.** Seventeen records forming eight groups were identical across all 31 substantive fields. All were retained for the primary analysis; a sensitivity analysis removing the nine redundant copies is reported.

5. **Prior-diagnosis harmonization.** The global diagnosis item and the specific-diagnosis checklist disagreed in 23 records (11 global-negative with a specific diagnosis; 12 global-positive with none, of which five carried explanatory free text). A harmonized variable was defined as affirmative on either source, yielding 399 positives. A sensitivity analysis using the unharmonized global item is reported.

6. **No imputation was performed** because the dataset contained no missing values.

Table S2. Hyperparameter search spaces and configurations selected during nested cross-validation

Model	Search space	Most frequently selected configuration (Model B)	Selected in n of 25 outer folds	Distinct configurations selected
Logistic regression	No tuning parameters (unpenalized, standardized inputs)	Not applicable	25	1
Elastic-net logistic regression	C in {0.01, 0.1, 1.0}; l1_ratio = 0.5; saga solver	C = 1.0, l1_ratio = 0.5	15	2
Random forest	n_estimators = 300; max_depth in {4, None}; min_samples_leaf in {10, 50}; max_features in {sqrt, 0.5}	max_depth = None, max_features = sqrt, min_samples_leaf = 50	11	4
Gradient boosting (XGBoost)	n_estimators = 300; max_depth in {2, 3}; learning_rate = 0.05; subsample = 0.8; colsample_bytree = 0.8; min_child_weight in {5, 20}; reg_lambda in {1.0, 5.0}	max_depth = 2, min_child_weight = 5, reg_lambda = 5.0, learning_rate = 0.05, n_estimators = 300, subsample = 0.8, colsample_bytree = 0.8	12	7
Decision tree	max_depth in {2, 3, 4, 5}; min_samples_leaf in {20, 50}	max_depth = 5, min_samples_leaf = 50	12	6
Support vector machine (RBF kernel, Platt scaling)	C in {0.1, 1.0, 10.0}; gamma in {scale, 0.01}; standardized inputs	C = 0.1, gamma = 0.01	25	1
Neural network (multilayer perceptron)	hidden layers in {(8), (16, 8)}; alpha (L2) in {0.001, 0.1}; ReLU; Adam; early stopping (15% validation, 20 epochs patience); standardized inputs	alpha = 0.001, hidden_layer_sizes = (16, 8)	12	4

For Model A the corresponding modal configurations were: elastic net, C = 1.0 (19 of 25 folds); random forest, max_depth = 4, max_features = sqrt, min_samples_leaf = 10 (15 of 25); gradient boosting, max_depth = 2, min_child_weight = 5, reg_lambda = 5.0 (11 of 25); decision tree, max_depth = 4, min_samples_leaf = 20 (9 of 25); support vector machine, C = 0.1, gamma = 0.01 (12 of 25); neural network, alpha = 0.1, hidden_layer_sizes

= (16, 8) (12 of 25). Selection was performed by inner three-fold stratified cross-validation optimizing AUROC, entirely within each outer training fold.

Table S3. Cross-validation performance by repetition (Model B)

Model	Repetition	AUROC	AUPRC	Brier
Logistic regression	1	0.726	0.706	0.207
Logistic regression	2	0.727	0.705	0.208
Logistic regression	3	0.725	0.706	0.207
Logistic regression	4	0.725	0.705	0.208
Logistic regression	5	0.722	0.698	0.209
Elastic-net logistic regression	1	0.726	0.706	0.207
Elastic-net logistic regression	2	0.727	0.705	0.208
Elastic-net logistic regression	3	0.725	0.705	0.208
Elastic-net logistic regression	4	0.724	0.704	0.208
Elastic-net logistic regression	5	0.721	0.699	0.209
Random forest	1	0.722	0.690	0.210
Random forest	2	0.715	0.685	0.211
Random forest	3	0.719	0.690	0.211
Random forest	4	0.722	0.689	0.210
Random forest	5	0.719	0.691	0.211
Gradient boosting (XGBoost)	1	0.724	0.709	0.207
Gradient boosting (XGBoost)	2	0.722	0.704	0.208
Gradient boosting (XGBoost)	3	0.724	0.708	0.207
Gradient boosting (XGBoost)	4	0.725	0.711	0.207
Gradient boosting (XGBoost)	5	0.718	0.693	0.210
Decision tree	1	0.693	0.665	0.216
Decision tree	2	0.693	0.657	0.218
Decision tree	3	0.697	0.667	0.217
Decision tree	4	0.700	0.667	0.215
Decision tree	5	0.690	0.651	0.218
Support vector machine	1	0.719	0.700	0.210
Support vector machine	2	0.718	0.697	0.211
Support vector machine	3	0.720	0.702	0.210
Support vector machine	4	0.718	0.698	0.210
Support vector machine	5	0.720	0.697	0.210
Neural network (MLP)	1	0.699	0.667	0.217
Neural network (MLP)	2	0.715	0.685	0.211
Neural network (MLP)	3	0.707	0.677	0.214
Neural network (MLP)	4	0.709	0.681	0.215
Neural network (MLP)	5	0.701	0.664	0.216

Each repetition provides one complete set of out-of-fold predictions covering all 1838 participants. Fold-level summaries (mean, standard deviation and 95% confidence interval across folds) are given in Table S10.

Table S4. Sensitivity analyses

Analysis	n	Outcome prevalence (%)	AUROC Model A (LR)	AUROC Model B (LR)	AUROC Model B (XGBoost)	Brier Model B (XGBoost)
Primary endpoint (at least 2 of 4 symptoms)	1838	45.7	0.664	0.727	0.725	0.206
S1: at least 3 of 4 symptoms	1838	24.6	0.659	0.741	0.737	0.156
S2: strict reflux definition, at least 2 of 4	1838	30.9	0.667	0.745	0.740	0.177
S3: self-rated digestive health below "good"	1838	39.3	0.673	0.718	0.718	0.204
S4: redundant identical records removed	1829	45.8	0.663	0.726	0.723	0.207
S5: records with out-of-window dates removed	1836	45.6	0.664	0.727	0.724	0.206
S6: unharmonized prior-diagnosis item	1838	45.7	0.664	0.725	0.724	0.206

Sensitivity analyses used three repetitions of stratified five-fold cross-validation with the modal hyperparameter configuration, which explains negligible differences from the corresponding values in the main analysis (for example 0.727 versus 0.726 for Model B logistic regression on the primary endpoint). Gradient-boosting

values were re-computed when the pipeline was re-executed for the revision; logistic regression values were reproduced exactly. All estimates are internal. LR, logistic regression.

Table S5. Extended subgroup performance

See Table 5 of the main text, which contains AUPRC, Brier score, calibration intercept and calibration slope for every subgroup, and Table 6, which reports Model A and Model B discrimination by previous digestive diagnosis and by access-to-care proxies. Subgroups with fewer than 10 events or 10 non-events were not evaluated. No department was excluded on that basis, but El Paraíso (n = 53) is reported with an explicit stability caveat.

S5a. Illustrative operating characteristics at higher sensitivity

Model	Target sensitivity	Threshold	Sensitivity	Specificity	PPV	NPV	Proportion flagged
Model A, logistic regression	0.80	0.362	0.800	0.374	0.518	0.689	0.706
Model A, logistic regression	0.85	0.338	0.850	0.304	0.507	0.706	0.767
Model A, logistic regression	0.90	0.308	0.900	0.229	0.496	0.732	0.830
Model B, logistic regression	0.80	0.327	0.800	0.457	0.554	0.731	0.661
Model B, logistic regression	0.85	0.303	0.850	0.370	0.532	0.745	0.731
Model B, logistic regression	0.90	0.274	0.900	0.268	0.508	0.761	0.809
Model B, gradient boosting	0.80	0.328	0.800	0.461	0.555	0.732	0.658
Model B, gradient boosting	0.85	0.299	0.850	0.377	0.534	0.749	0.727
Model B, gradient boosting	0.90	0.261	0.900	0.258	0.505	0.754	0.814

These thresholds were located on the out-of-fold predictions rather than within training folds. They are presented to illustrate the sensitivity-specificity trade-off available at this level of discrimination and must not be treated as independently validated operating points. The nested (training-fold) 80%-sensitivity threshold for Model B logistic regression, which replaces the illustrative value in the main text, is reported in Table 8 of the main text.

Table S6. TRIPOD+AI checklist mapped to manuscript sections

Item numbering follows the TRIPOD+AI checklist (Collins et al., BMJ 2024). Items marked "not applicable" or "partly" are also summarized in Section 2.1 of the main text.

Item	Topic	Location in manuscript	Addressed
1	Title	Title (concurrent classification model, target population, outcome)	Yes
2	Abstract	Abstract	Yes
3a	Background: healthcare context and rationale	Introduction, paragraphs 1 and 2	Yes
3b	Background: target population and intended use	Introduction, final two paragraphs	Yes
3c	Background: known biases and inequalities	Introduction, paragraph 3	Yes
4	Objectives	Introduction, final paragraphs	Yes
5a	Data sources	Methods 2.1, 2.3	Yes
5b	Data collection dates	Methods 2.1	Yes
6a	Setting and centres	Methods 2.1, 2.2	Yes
6b	Eligibility criteria	Methods 2.2	Yes
6c	Treatments received	Not applicable (no intervention)	Not applicable
7	Data preparation and quality checks	Methods 2.6; Table S1	Yes
8a	Outcome definition and timing	Methods 2.4	Yes
8b	Outcome assessment and qualifications	Methods 2.3 (single self-report instrument)	Partly
8c	Blinding of outcome assessment	Not possible; same interview (Discussion, limitations)	Partly
9a	Choice of predictors	Methods 2.5	Yes
9b	Predictor definition and measurement	Methods 2.5; Table S1	Yes
9c	Blinding of predictor assessment	Not possible; same interview	Partly
10	Sample size	Methods 2.5 (events per parameter)	Yes
11	Missing data	Methods 2.6 (no missing data)	Yes
12a	Data use in development and evaluation	Methods 2.9	Yes
12b	Predictor handling	Methods 2.5, 2.8	Yes
12c	Model type, building, hyperparameter tuning, internal validation	Methods 2.8, 2.9; Table S2	Yes

Item	Topic	Location in manuscript	Addressed
12d	Heterogeneity across clusters	Methods 2.9, 2.10 (grouped validation, cluster bootstrap)	Yes
12e	Performance measures and model comparison	Methods 2.10	Yes
12f	Model updating or recalibration	Methods 2.11 (recalibration-in-the-large, exploratory)	Yes
12g	Model specification for external validation	Not applicable (development study)	Not applicable
13	Class imbalance	Methods 2.8 (none applied)	Yes
14	Fairness	Methods 2.11; Results 3.6; Tables 5, 6, 8	Yes
15	Model output and thresholds	Methods 2.10; Table 8	Yes
16	Development versus evaluation data differences	Not applicable (single dataset, internal validation)	Not applicable
17	Ethical approval	Methods 2.14; IRB statement	Yes
18a	Funding	Funding statement	Yes
18b	Conflicts of interest	Conflicts of Interest	Yes
18c	Protocol	Protocol available from corresponding author	Partly
18d	Registration	Not registered	Partly
18e	Data sharing	Data Availability Statement	Yes
18f	Code sharing	File S1	Yes
19	Patient and public involvement	Community leaders facilitated access; no formal involvement in design	Partly
20a	Participant flow	Results 3.1; Figure 1 (approach and refusal counts pending from field team)	Partly
20b	Participant characteristics	Table 1	Yes
20c	Comparison of development and evaluation data	Not applicable	Not applicable
21	Model development (participants, events)	Results 3.1, 3.3; Table 3	Yes
22	Model specification	Table 4; Table S9 (full equation)	Yes
23a	Performance with confidence intervals	Tables 3, 7; Figures 2, 3	Yes
23b	Performance in key subgroups	Tables 5, 6, 8	Yes
24	Model updating results	Not applicable	Not applicable
25	Interpretation	Discussion, paragraphs 1 to 7	Yes
26	Limitations	Discussion, limitations paragraph	Yes
27a	Usability: handling poor-quality or unavailable input	Discussion (Model A when previous history unavailable)	Yes
27b	Usability: user interaction and expertise	Discussion (paper or spreadsheet equation; Table S9)	Partly
27c	Future research	Discussion, final paragraph	Yes

Table S7. STROBE cross-sectional checklist mapped to manuscript sections

Item	Topic	Location in manuscript	Addressed
1a	Design indicated in title or abstract	Title; Abstract	Yes
1b	Informative abstract	Abstract	Yes
2	Scientific background and rationale	Introduction	Yes
3	Objectives and hypotheses	Introduction, final paragraphs	Yes
4	Study design	Methods 2.1	Yes
5	Setting, locations, dates	Methods 2.1, 2.2	Yes
6a	Eligibility criteria and selection	Methods 2.2	Yes
7	Variables defined	Methods 2.4, 2.5; Table S1	Yes
8	Data sources and measurement	Methods 2.3	Yes
9	Bias	Methods 2.2, 2.6; Discussion, limitations	Yes
10	Study size	Methods 2.2, 2.5; Results 3.1	Yes
11	Quantitative variables	Methods 2.5; Table S1	Yes
12a to 12e	Statistical methods, subgroups, missing data, sampling strategy, sensitivity analyses	Methods 2.6 to 2.12	Yes
13a	Numbers at each stage	Results 3.1; Figure 1 (approached and declining counts pending)	Partly
13b	Reasons for non-participation	Not recorded	Partly
13c	Flow diagram	Figure 1	Yes
14a to 14b	Descriptive data, missing data	Table 1; Methods 2.6	Yes
15	Outcome data	Results 3.2; Table 2	Yes
16a to 16c	Main results, adjustment, absolute risks	Tables 3 to 8; Results 3.3 to 3.6	Yes
17	Other analyses	Results 3.6; Tables S4, S8	Yes
18	Key results	Discussion, paragraph 1; Conclusions	Yes
19	Limitations	Discussion, limitations paragraph	Yes
20	Interpretation	Discussion	Yes
21	Generalizability	Discussion; Methods 2.2	Yes
22	Funding	Funding statement	Yes

Table S8. Models for individual symptom indicators and ordinal model for symptom count

Endpoint	Prevalence	AUROC Model A (95% CI)	AUROC Model B (95% CI)	Calibration slope (Model B)
Abdominal pain	0.324	0.619 (0.590 to 0.648)	0.677 (0.648 to 0.702)	0.953
Heartburn	0.310	0.640 (0.613 to 0.669)	0.714 (0.686 to 0.741)	0.956
Any reflux	0.553	0.643 (0.619 to 0.669)	0.690 (0.666 to 0.712)	0.941
Frequent or constant reflux	0.106	0.686 (0.650 to 0.725)	0.761 (0.726 to 0.796)	0.943
Post-prandial bloating	0.319	0.612 (0.585 to 0.640)	0.669 (0.642 to 0.695)	0.930
Primary outcome (at least 2 of 4)	0.457	0.663 (0.639 to 0.689)	0.726 (0.701 to 0.748)	0.955
At least 3 of 4	0.246	0.660 (0.629 to 0.691)	0.742 (0.714 to 0.767)	0.961

Predictor	Pain	Heartburn	Any reflux	Frequent reflux	Bloating	Count (ordinal)
Age, per 10 years	1.11 (1.00 to 1.23)	1.18 (1.07 to 1.31)	1.16 (1.06 to 1.28)	1.22 (1.04 to 1.43)	1.03 (0.93 to 1.14)	1.16 (1.07 to 1.26)
Female sex	1.11 (0.88 to 1.40)	1.44 (1.13 to 1.83)	0.90 (0.73 to 1.12)	1.91 (1.31 to 2.79)	1.37 (1.09 to 1.73)	1.17 (0.97 to 1.40)
Education, per category	0.93 (0.87 to 1.00)	0.89 (0.83 to 0.96)	1.14 (1.06 to 1.23)	0.96 (0.86 to 1.07)	1.04 (0.96 to 1.11)	1.02 (0.96 to 1.08)
Daily fibre intake	0.86 (0.69 to 1.07)	0.82 (0.65 to 1.03)	0.83 (0.67 to 1.03)	0.64 (0.47 to 0.89)	0.75 (0.61 to 0.94)	0.78 (0.65 to 0.94)
At least 8 glasses of water	0.74 (0.60 to 0.92)	0.87 (0.69 to 1.08)	0.93 (0.76 to 1.14)	1.02 (0.73 to 1.42)	0.81 (0.65 to 1.00)	0.82 (0.69 to 0.97)
Physical activity	0.81 (0.65 to 1.00)	1.05 (0.85 to 1.32)	0.68 (0.56 to 0.84)	0.86 (0.61 to 1.20)	0.83 (0.67 to 1.03)	0.77 (0.64 to 0.91)
Current smoking	1.54 (1.08 to 2.20)	1.74 (1.21 to 2.51)	1.89 (1.26 to 2.82)	1.30 (0.81 to 2.09)	1.59 (1.12 to 2.27)	1.90 (1.41 to 2.56)
Habitual alcohol	1.81 (1.34 to 2.45)	1.81 (1.33 to 2.47)	1.72 (1.25 to 2.36)	2.51 (1.66 to 3.78)	1.68 (1.24 to 2.27)	2.01 (1.56 to 2.57)
Previous diagnosis	3.42 (2.69 to 4.35)	4.27 (3.34 to 5.45)	4.06 (3.07 to 5.38)	4.44 (3.20 to 6.14)	2.99 (2.36 to 3.80)	5.22 (4.21 to 6.47)

Upper panel: five-fold cross-validation repeated five times, logistic regression. Lower panel: adjusted odds ratios (95% CI) from multivariable logistic models for each symptom and from a proportional-odds ordinal logistic model for the 0 to 4 symptom count, all with the nine Model B predictors. The cumulative odds ratios for the previous-diagnosis item at cut-points 1, 2, 3 and 4 symptoms were 8.0, 5.1, 5.0 and 5.3, and those of the other predictors were similarly stable, supporting the proportional-odds approximation.

Table S9. Full Model B logistic regression equation (fitted to all 1838 participants)

Term	Coefficient (log-odds)	Standard error	Coding
Intercept	−0.9081	0.2243	Intercept
Age	0.0185	0.0049	Years (12 to 49)
Female sex	0.1888	0.1145	1 = female
Education	0.0137	0.0364	0 to 6
Daily fibre intake	−0.2468	0.1100	1 = yes
At least 8 glasses of water	−0.2987	0.1075	1 = yes
Physical activity	−0.3851	0.1058	1 = yes
Current smoking	0.6877	0.1966	1 = yes
Habitual alcohol	0.7321	0.1587	1 = yes
Previous diagnosis	1.6356	0.1369	1 = yes

Predicted probability = 1 / (1 + exp(−LP)), where LP is the intercept plus the sum of each coefficient multiplied by the corresponding predictor value in its original units. Example: a 35-year-old woman with incomplete secondary education (3), daily fibre intake, no adequate water intake, no regular physical activity, no smoking, habitual alcohol use and a previous digestive diagnosis has LP = 2.092 and a predicted probability of 0.890. Coefficients come from the model fitted to all participants; performance estimates come from nested cross-validation.

Table S10. Fold-level performance under five-fold and ten-fold nested cross-validation, with apparent (training-fold) performance

Model	CV scheme	Pooled AU-ROC	Fold mean AU-ROC (SD)	95% CI across folds	Fold range	Training-fold AU-ROC	Optimism	Calibration slope
**Model B**								
Logistic regression	5-fold x 5	0.726	0.726 (0.026)	0.715 to 0.736	0.685 to 0.796	0.734	0.008	0.955
	10-fold x 5	0.726	0.726 (0.036)	0.716 to 0.736	0.658 to 0.844	0.734	0.007	0.958
Elastic-net logistic regression	5-fold x 5	0.725	0.726 (0.025)	0.715 to 0.736	0.685 to 0.794	0.734	0.008	0.981
	10-fold x 5	0.726	0.726 (0.035)	0.716 to 0.736	0.658 to 0.843	0.733	0.008	0.985
Random forest	5-fold x 5	0.723	0.722 (0.023)	0.713 to 0.732	0.675 to 0.783	0.770	0.048	1.254
	10-fold x 3	0.720	0.722 (0.031)	0.710 to 0.733	0.661 to 0.774	0.761	0.040	1.273
Gradient boosting (XGBoost)	5-fold x 5	0.725	0.723 (0.025)	0.713 to 0.733	0.681 to 0.770	0.773	0.049	0.930
	10-fold x 5	0.725	0.724 (0.034)	0.715 to 0.734	0.663 to 0.804	0.769	0.045	0.926
Decision tree	5-fold x 5	0.704	0.695 (0.024)	0.685 to 0.705	0.656 to 0.746	0.731	0.036	0.927
	10-fold x 5	0.701	0.695 (0.031)	0.686 to 0.704	0.618 to 0.787	0.732	0.037	0.886
Support vector machine	5-fold x 5	0.720	0.724 (0.025)	0.714 to 0.734	0.686 to 0.784	0.730	0.006	0.917
	10-fold x 5	0.720	0.724 (0.036)	0.714 to 0.734	0.666 to 0.842	0.730	0.005	0.919
Neural network (MLP)	5-fold x 5	0.713	0.707 (0.027)	0.696 to 0.718	0.664 to 0.760	0.725	0.018	1.052
	10-fold x 5	0.713	0.711 (0.036)	0.700 to 0.721	0.646 to 0.825	0.723	0.013	1.045
**Model A**								
Logistic regression	5-fold x 5	0.663	0.663 (0.030)	0.651 to 0.676	0.595 to 0.726	0.672	0.009	0.939
	10-fold x 5	0.663	0.663 (0.041)	0.651 to 0.675	0.573 to 0.772	0.672	0.009	0.939
Elastic-net logistic regression	5-fold x 5	0.663	0.663 (0.030)	0.651 to 0.675	0.595 to 0.726	0.672	0.009	0.957
	10-fold x 5	0.663	0.663 (0.041)	0.651 to 0.675	0.573 to 0.771	0.672	0.009	0.954
Random forest	5-fold x 5	0.660	0.660 (0.026)	0.649 to 0.671	0.617 to 0.727	0.714	0.054	1.290
	10-fold x 3	0.659	0.660 (0.042)	0.645 to 0.676	0.563 to 0.740	0.704	0.043	1.309
Gradient boosting (XGBoost)	5-fold x 5	0.661	0.659 (0.029)	0.647 to 0.670	0.611 to 0.718	0.723	0.064	0.869
	10-fold x 5	0.663	0.662 (0.040)	0.651 to 0.673	0.567 to 0.777	0.716	0.054	0.884
Decision tree	5-fold x 5	0.643	0.629 (0.026)	0.618 to 0.640	0.575 to 0.697	0.678	0.049	0.865
	10-fold x 5	0.641	0.632 (0.040)	0.621 to 0.643	0.535 to 0.725	0.677	0.045	0.811
Support vector machine	5-fold x 5	0.650	0.654 (0.030)	0.641 to 0.666	0.600 to 0.729	0.670	0.016	0.988
	10-fold x 5	0.654	0.656 (0.043)	0.644 to 0.668	0.560 to 0.758	0.667	0.011	0.984
Neural network (MLP)	5-fold x 5	0.654	0.646 (0.030)	0.634 to 0.658	0.583 to 0.710	0.670	0.024	0.991
	10-fold x 5	0.656	0.646 (0.051)	0.632 to 0.661	0.527 to 0.784	0.664	0.018	1.025

Fold-level confidence intervals are t-based intervals across held-out folds and are descriptive, because folds share training data. Optimism is the mean apparent (training-fold) AUROC minus the mean held-out AUROC. Random forest was repeated three times under ten-fold cross-validation because of computational cost.

Table S11. Additional classifiers: decision tree, support vector machine and neural network

Model	AUROC (95% CI)	AUPRC	Brier	Calibration slope	Sensitivity	Specificity	F1	Youden threshold
Model A, Logistic regression	0.663 (0.639 to 0.688)	0.626	0.227	0.939	0.517	0.706	0.554	0.476
Model A, Decision tree	0.643 (0.618 to 0.669)	0.598	0.231	0.865	0.473	0.723	0.525	0.476
Model A, Support vector machine	0.650 (0.625 to 0.675)	0.614	0.231	0.988	0.443	0.760	0.512	0.452
Model A, Neural network (MLP)	0.654 (0.630 to 0.679)	0.608	0.230	0.991	0.583	0.638	0.580	0.453
Model B, Logistic regression	0.726 (0.702 to 0.748)	0.705	0.208	0.955	0.555	0.798	0.618	0.475
Model B, Decision tree	0.704 (0.679 to 0.728)	0.674	0.212	0.927	0.488	0.854	0.587	0.528
Model B, Support vector machine	0.720 (0.697 to 0.743)	0.701	0.210	0.917	0.525	0.831	0.608	0.492
Model B, Neural network (MLP)	0.713 (0.689 to 0.737)	0.682	0.212	1.052	0.561	0.792	0.620	0.488

All models were tuned and evaluated under the same five-fold nested cross-validation repeated five times. Logistic regression is included for reference.

Table S12. PROBAST+AI self-assessment (model development)

Domain	Key considerations	Concern for quality / risk of bias	Applicability concern
Participants and data sources	Community convenience sample from 85 communities; eligibility explicit; counts approached and declining not recorded; no household identifier	High (selection cannot be fully evaluated)	Moderate: ages 12 to 49 years without known organic disease only
Predictors	Nine predictors defined in advance, collected with one standardized questionnaire, obtainable in primary care; self-reported; assessed at the same interview as the outcome	Low to unclear (self-report; no blinding possible)	Low
Outcome	Study-defined composite of four self-reported symptoms; no clinical reference standard; same instrument as predictors	Unclear (no validated reference standard)	Moderate: symptom burden, not a diagnosis
Analysis	840 events for 9 parameters; no missing data; no data-driven predictor selection; nested cross-validation, grouped and cluster-aware uncertainty, calibration, decision curves, fairness and paired comparisons reported; no external validation	Low for internal validity; external validity not assessed	Not applicable
Overall	Model suitable for external validation, not for implementation	High (driven by participant and outcome domains)	Moderate

Table S13. Participant-level versus community-level (cluster) bootstrap 95% confidence intervals for principal models

Model	AUROC	AUROC CI, participant	AUROC CI, community	Brier CI, participant	Brier CI, community	Calibration slope CI, community
Model A, Logistic regression	0.663	0.639 to 0.688	0.605 to 0.717	0.221 to 0.234	0.214 to 0.241	0.637 to 1.264
Model B, Logistic regression	0.726	0.702 to 0.748	0.675 to 0.772	0.200 to 0.216	0.193 to 0.223	0.755 to 1.161
Model B, Elastic-net logistic regression	0.725	0.702 to 0.748	0.675 to 0.772	0.200 to 0.216	0.193 to 0.223	0.775 to 1.194
Model B, Random forest	0.723	0.699 to 0.746	0.669 to 0.773	0.203 to 0.216	0.196 to 0.224	0.957 to 1.561
Model B, Gradient boosting (XGBoost)	0.725	0.700 to 0.748	0.676 to 0.773	0.198 to 0.215	0.191 to 0.223	0.739 to 1.138

Community-level intervals resample the 85 communities with replacement (2000 resamples), preserving within-community correlation.

Figure S1. Calibration plots for all candidate models

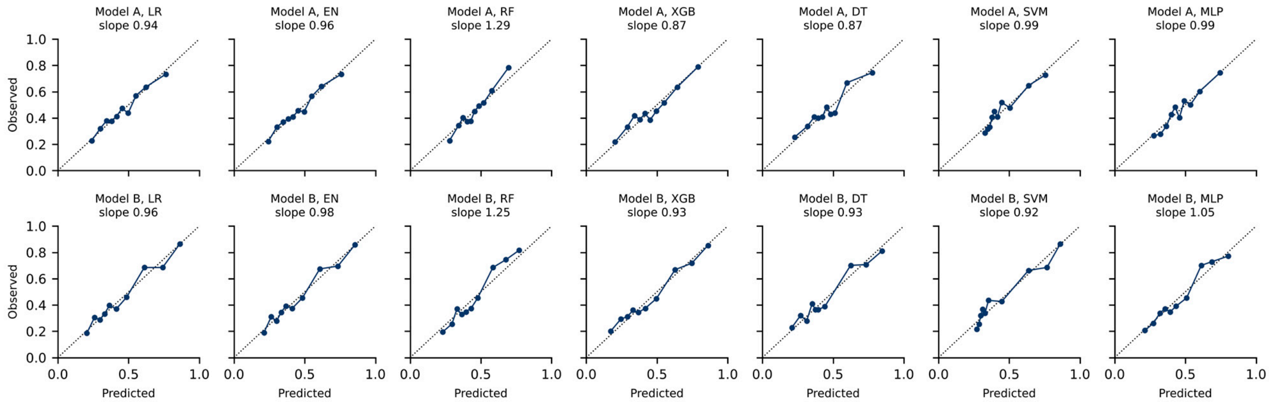

Decile-binned observed proportion against mean predicted probability for all seven Model A and Model B algorithms, using out-of-fold predictions. Generated by analysis_pipeline.py. The plot demonstrates the systematic compression of random forest predictions toward the sample prevalence (calibration slope 1.25 to 1.29) and the close agreement of the two regression models with the identity line.

Figure S2. SHAP dependence plots

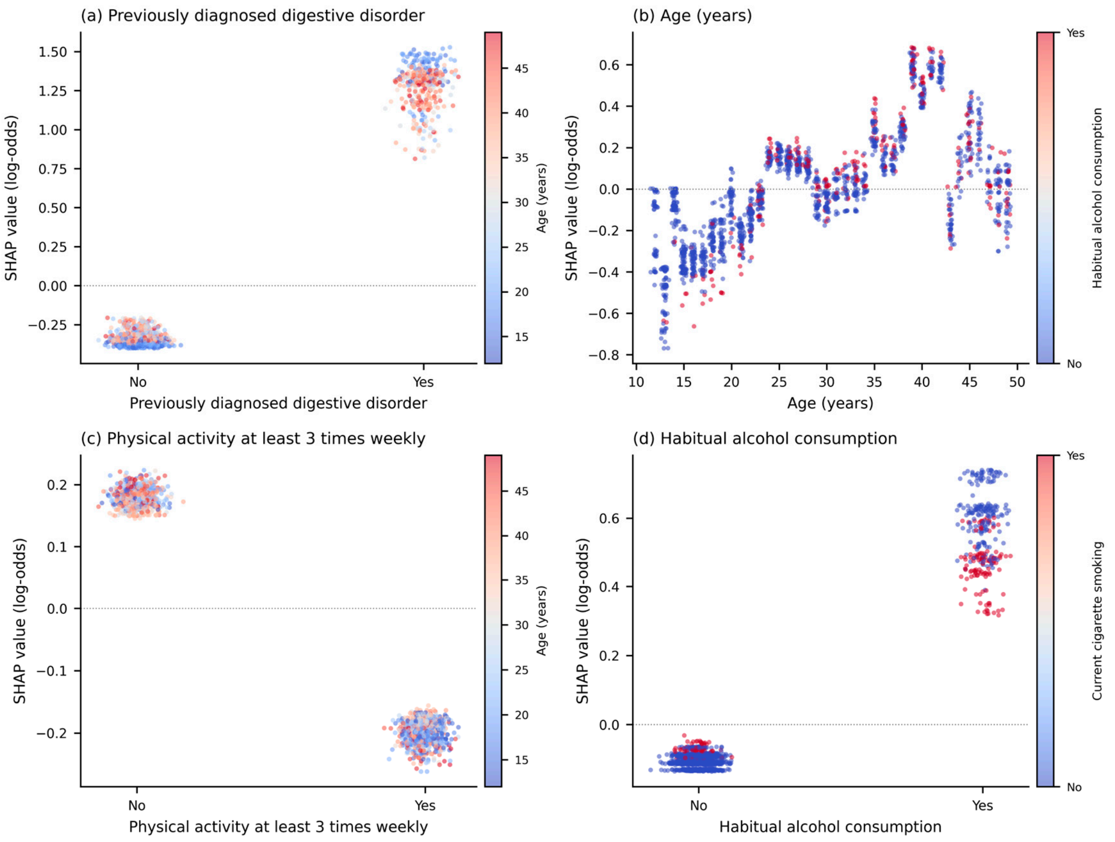

Dependence plots for the four highest-ranked variables in the Model B gradient-boosting classifier (previous digestive diagnosis, age, physical activity and alcohol consumption), showing the SHAP attribution against the feature value with colouring by the strongest interacting feature. Spearman correlations between feature value and SHAP attribution were: age 0.699; female sex 0.825; educational attainment 0.344; daily fibre intake −0.827; adequate water intake −0.857; physical activity −0.865; current smoking 0.526; habitual alcohol consumption 0.640; previous digestive diagnosis 0.714.

Figure S3. Distribution of fold-level AUROC under five-fold and ten-fold nested cross-validation

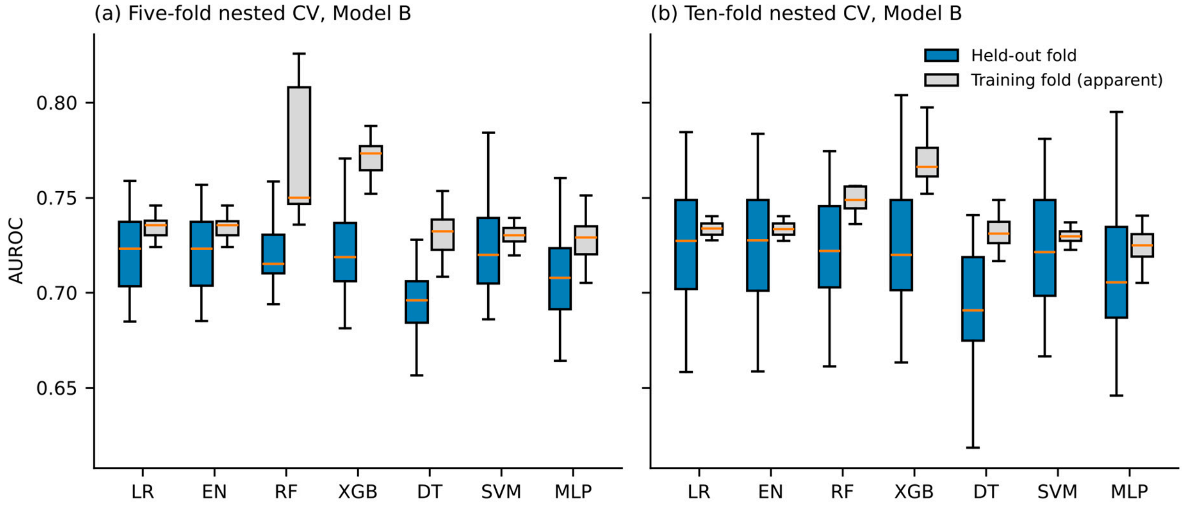

Held-out (blue) and apparent training-fold (grey) AUROC for the seven Model B algorithms under (a) five-fold cross-validation repeated five times and (b) ten-fold cross-validation repeated five times (three for random forest). LR, logistic regression; EN, elastic net; RF, random forest; XGB, gradient boosting; DT, decision tree; SVM, support vector machine; MLP, multilayer perceptron.
